# i-Motif formation and spontaneous deletions in human cells

**DOI:** 10.1093/nar/gkac158

**Published:** 2022-03-07

**Authors:** Marianna Martella, Flavia Pichiorri, Rupesh V Chikhale, Mahmoud A S Abdelhamid, Zoë A E Waller, Steven S Smith

**Affiliations:** Judy and Bernard Briskin Center for Multiple Myeloma Research, City of Hope, 1500 E. Duarte Rd., Duarte, CA 91010, USA; Judy and Bernard Briskin Center for Multiple Myeloma Research, City of Hope, 1500 E. Duarte Rd., Duarte, CA 91010, USA; School of Pharmacy, University of East Anglia, Norwich Research Park, Norwich NR4 7TJ, UK; UCL School of Pharmacy, 29-39 Brunswick Square, London WC1N 1AX, UK; School of Pharmacy, University of East Anglia, Norwich Research Park, Norwich NR4 7TJ, UK; School of Pharmacy, University of East Anglia, Norwich Research Park, Norwich NR4 7TJ, UK; UCL School of Pharmacy, 29-39 Brunswick Square, London WC1N 1AX, UK; Department of Hematologic Malignancies Translational Science, City of Hope, 1500 E. Duarte Rd., Duarte, CA 91010, USA; Beckman Research Institute of the City of Hope, 1500 E. Duarte Rd., Duarte, CA 91010, USA

## Abstract

Concatemers of d(TCCC) that were first detected through their association with deletions at the RACK7 locus, are widespread throughout the human genome. Circular dichroism spectra show that d(GGGA)_*n*_ sequences form G-quadruplexes when *n* > 3, while i-motif structures form at d(TCCC)_*n*_ sequences at neutral pH when *n* ≥ 7 *in vitro*. In the PC3 cell line, deletions are observed only when the d(TCCC)_*n*_ variant is long enough to form significant levels of unresolved i-motif structure at neutral pH. The presence of an unresolved i-motif at a representative d(TCCC)_*n*_ element at RACK7 was suggested by experiments showing that that the region containing the d(TCCC)_9_ element was susceptible to bisulfite attack in native DNA and that d(TCCC)_9_ oligo formed an i-motif structure at neutral pH. This in turn suggested that that the i-motif present at this site in native DNA must be susceptible to bisulfite mediated deamination even though it is a closed structure. Bisulfite deamination of the i-motif structure in the model oligodeoxynucleotide was confirmed using mass spectrometry analysis. We conclude that while G-quadruplex formation may contribute to spontaneous mutation at these sites, deletions actually require the potential for i-motif to form and remain unresolved at neutral pH.

## INTRODUCTION

Regions of GC Skew in eukaryotic genomes, where dG residues are generally confined to one strand and dC residues are confined to the other, are prone to the formation of several types of non-B DNA structure. Among the more common of these are regions that conform to a general sequence motif: d(G_3+_N_1–7_G_3+_N_1–7_G_3+_N_1–7_G_3+_). This motif has been used to detect genomic sites with potential to fold into G-quadruplex structures on the G-rich strand in regions of GC Skew (1). Since G-quadruplex structures are strongly stabilized by the presence of potassium (2), they are favored under physiological conditions. Nevertheless other alternative DNA structures such as triplexes (3) and i-motifs (4) can also form at sites conforming to this general sequence motif.

Genomic sequence comparisons across Eukarya show that sequences conforming to this motif have increased in frequency during the evolution of ever more complex eukaryotic organisms (5,6) suggesting that they are under positive selective pressure and may serve one or more biological functions. Since they are often present in promotor regions (7) there has been considerable interest in the possibility that they play roles in transcription and other epigenetic phenomena. Evidence in support of these ideas has been obtained in a number of studies. Triplex formation at the MYC promoter has been implicated in transcription regulation at this gene (3), and i-motif formation at the BCL2 gene has been shown to play a role in transcription at that locus (8). Moreover, formation of stable structures at these sequences has been shown to promote methylation in their vicinity (9) and disrupt the formation of repressive chromatin structures (10). G-quadruplexes, for example may influence DNA methylation patterns via the direct inhibition of DNA methyltransferase I (11). It has also been shown that G-quadruplex formation on the G-rich strand in a region of GC Skew may indirectly influence DNA methylation patterning by stabilizing R-loop RNA:DNA hybrids on the C-rich DNA strand, since RNA:DNA hybrids have also been shown *in vitro* to inhibit DNA methyltransferase I (12,13).

In normal cells, G-quadruplex formation is likely to be transient, given the constellation of G-quadruplex resolvases known to be expressed in normal mammalian cells. Among these are the RecQ helicases encoded by the BLM (14) and WRN genes (15) and the helicase encoded by the Fanconi Anemia locus FANCJ (16). Further, during DNA replication in normal cells the Y-polymerase family genes (REV1, POLK and POLH) express polymerases capable of replication through G-quadruplex DNA. Rev1 actually disrupts G residues in the quadruplex and inserts a dC residue on the nascent strand (17) while, Pol κ and Pol η also appear to function in replicating through G-quadruplex forming sequences (18). Even so, replication through extended regions of G-quadruplex potential like the d(GGC)_*n*_ repeat at FRAXA in disease carriers appears to be able to induce replication fork collapse and chromosome breaks and subsequent deletion and insertion prone repair (19).

In the most extensive study of quadruplex-linked replication impediments to date, the dog-1 mutation in *Caenorhabditis elegans* was shown to promote genome wide deletions confined to oligo dG sequences (20,21). The mutation at dog-1 knocks out the *C. elegans* homolog of human FANCJ, which is known to resolve G-quadruplex sequences (16), thus implicating unresolved G-quadruplex sequences as replication impediments at multiple genomic locations. The deletions in *C. elegans* were shown to occur 5′- to the oligo dG strand (i.e. the G-rich strand), and often deleted much of the oligo dG element itself at each site (20). Deletion frequency also increased with the length of the oligo dG element (20).

Interestingly, the oligo dC strand has been shown to form an i-motif quadruplex at neutral pH *in vitro* (22). These sequences form optimally when they conform to a (4*n*– 1) length rule for *n* > 2 (22). That is to say, the potential for i-motif structure at pH 7 *in vitro* is greatest at lengths of 11, 15, 19 and 23 dC residues, since the pH_T_ (i.e. the pH at which half the population of sequence adopts an i-motif) is 7.2 at sequence lengths conforming to this (4*n* –1) rule. Data from the dog-1 experiments shows that at lengths of 15 and 19 the deletion frequency appears to be suppressed but not at sequences of greater length (20). For short sequences of this type this is consistent with evidence showing that G-quadruplex and i-motif formation at shorter sequences can be mutually exclusive due to steric hindrance while other, often longer sequences, permit the formation of both structures offset from one another (23–25). Taken together, this suggests that i-motif formation in oligo dC at lengths of 15 and 19 suppresses G-quadruplex formation in *C. elegans* and thus hinders dog-1 induced deletions, while at longer d(G:C) sequences the formation of i-motifs does not prevent formation of G-quadruplexes resulting in dog-1 induced deletions. For this interpretation to be correct, *C. elegans* must either retain a repair system that resolves i-motif structures, or i-motif structures do not form replication impediments in *C. elegans*.

In previous work, we devised a method designed to clone sequences from stalled replication forks at sites in DNA where DNA polymerase and CMG helicase uncoupling is present prior to DNA isolation (Supplementary Figure S1). Double strand fragments originating near a putative CMG helicase halt site were often recovered associated with a non-B structure forming element generally detected within 1800 bp of the cloned sequence (26). One of the sequences detected in that screen, d(TCCC)_9_, was shown to be in an open conformation in PC3 cells as judged by bisulfite accessibility in native chromosomal DNA, and was characterized by short deletions in the PC3 prostate tumor cell line (26). Since the sequence was capable of forming an i-motif at neutral pH *in vitro*, this raised the possibility that the i-motif structure may form a replication impediment that is not suppressed in human prostate cancer cells. The absence of a functional p53 gene in the PC3 cell line is consistent with the possibility that i-motif suppression by DNA repair systems could be defective in this repair compromised cell line.

In this report we determined the tendency of the d(TCCC)_*n*_ sequences to from i-motif structures and the tendency of the d(GGGA)_*n*_ complementary sequence to form G-quadruplex at neutral pH *in vitro*. Since the d(TCCC)_*n*_ element occurs with different lengths at multiple chromosomal locations in the human genome we compared these *in vitro* results with the frequency of deletions at sites ranging from d(TCCC)_5_ to d(TCCC)_15_ observed in the PC3 cell line. We found that G-quadruplex formation *in vitro* was spontaneous at physiological pH over the range tested d(GGGA)_4_ to d(GGGA)_14_ while i-motif formation at neutral pH increased over the range d(TCCC)_5_ to d(TCCC)_15_. Deletion frequency in the PC3 cell line reached 100% of cloned representatives when the pH_T_ observed *in vitro* for i-motif formation exceeded pH 7. Moreover, deletions were confined 5′- to the d(TCCC)_n_ element (i.e. only on the C-rich strand) in each gene examined.

## MATERIALS AND METHODS

### Cell culture

Cell culture methods and preparation of DNA from the PC3 cell line were as previously described (27).

### DNA isolation

DNA isolation methods have been previously described (27).

### PCR methods

All PCR Primers were obtained from Integrated DNA Technologies (San Diego, CA).

For the region from the TAFA5 gene: The forward primer sequence was 5′-GCACTCAGCTCTCTGCTGTCCTTGCCTC-3′, the reverse primer sequence was: 5′-AGACCCTGATCCAGCCAGCCAAGCCCTC-3′. PCR reactions contained 12.5 μl 2X KAPA2G Fast Multiplex mix (Kapa Biosystems, Wilmington, MA), 1.25 μl of 10 μM stock of each primer (500 nM final concentration), 100 ng target DNA, made up to a final volume of 25 μl with Molecular Biology Water (Sigma). PCR cycling conditions were 95°C for 5 min, followed by 35 cycles of 95°C for 15 s, 56°C for 30 s, 72°C for 30 s followed by a final extension at 72°C for 26 s.

For the region from the BCR gene: The forward primer sequence was 5′-TTGCTCCTCTTATTTCCTAGCAGCAGATGT-3′, the reverse primer sequence was: 5′-CAGTAGGTGTCACATCAACTTCAAATGAAC-3′. PCR reactions contained 12.5 μl 2X KAPA2G Fast Multiplex mix (Kapa Biosystems, Wilmington, MA), 0.55 μl of 9 μM stock of each primer (198 nM final concentration), 200 ng target DNA, made up to a final volume of 25 μl with Molecular Biology Water (Sigma). PCR cycling conditions were 95°C for 5 min, followed by 35 cycles of 95°C for 15 s, 56°C for 30 s, 72°C for 30 s followed by a final extension at 72°C for 26 s.

For the region from the ABL1gene: The forward primer sequence was 5′-ATATACTTAGAGGTGGGCTGGTAAAG-3′, the reverse primer sequence was: 5′-CCATGTTAGGTCAGGTGCTCATGTCT-3′. PCR reactions contained 12.5 μl 2X KAPA2G Fast Multiplex mix (Kapa Biosystems, Wilmington, MA), 1.25 μl of 10 μM stock of each primer (500 nM final concentration), 100 ng target DNA, made up to a final volume of 25 μl with Molecular Biology Water (Sigma). PCR cycling conditions were 95°C for 3 min, followed by 35 cycles of 95°C for 15 s, 53°C for 30 s, 72°C for 30 s followed by a final extension at 72°C for 26 s.

For the region from the RACK 7 gene: The forward primer sequence was 5′-GGTTGGCCAGTTGTGTACCTCACAGTGG-3′; and the reverse primer sequence was 5′-GCTCCAGCCTGGGCAACAAAGCAACAGT-3′. PCR reactions contained 12.5 μl 2X KAPA2G Fast Multiplex mix (Kapa Biosystems, Wilmington, MA), 0.55 μl 9 μM stock of each primer (198 nM final concentration), 200 ng target DNA, made up to a final volume of 25 μl with Molecular Biology Water (Sigma). PCR cycling conditions were 95°C for 3 min, followed by 35 cycles of 95°C for 1 min, 60°C for 30 s, 72°C for 30 s followed by a final extension at 72°C for 26 s.

For the region from the PLA2G2A gene: The forward primer sequence was 5′-TAGAGGTGATGGTTGTACAACACTGTGAAT-3′; and the reverse primer sequence was 5′-AGAGCAAGACCCTGTCTTATTGTTGAACCG-3′. PCR reactions contained 12.5 μl 2X KAPA2G Fast Multiplex mix (Kapa Biosystems, Wilmington, MA), 0.55 μl of 9 μM stock of each primer (198 nM final concentration), 200 ng target DNA, made up to a final volume of 25 μl with Molecular Biology Water (Sigma). PCR cycling conditions were 95°C for 3 min, followed by 35 cycles of 95°C for 1 min, 63°C for 30 s, 72°C for 30 s followed by a final extension at 72°C for 26 s.

### Bisulfite mediated deamination of native genomic DNA

Genomic DNA isolated from the PC3 cell line was treated with bisulfite using the EZ methylation Kit (Zymo Research, Irvine CA). For these experiments, DNA was added to the conversion reagent without M-dilution buffer (a denaturant) or heat treatment in order to preserve the native structure present in the isolated DNA. The reaction mixture was incubated in the dark at 37°C for 16 h. Thereafter, the kit protocol including the desulfonation step was carried out in order to recover DNA for subsequent PCR amplification of the selected region in the RACK7 gene.

### *In vitro* analysis of d(TCCC)_*n*_ and d(GGGA)_*n*_ oligodeoxynucleotides

#### Oligonucleotides

were purchased from IDT (San Diego, CA) or Eurogentec (Liege, Belgium) and were HPLC purified. Solid DNA samples were initially dissolved as a stock solution in purified water (1 mM); further dilutions were carried out in the respective sodium cacodylate buffer. Samples were thermally annealed in a heat block at 95°C for 5 min and cooled slowly to room temperature overnight.

#### UV absorption spectroscopy

UV spectroscopy experiments were performed on a Cary 60 UV–Vis spectrometer (Agilent Technologies) and recorded using low volume masked quartz cuvettes (1 cm path length). ODNs were diluted to 2.5 μM in buffer at the desired pH. Samples (200 μl) were transferred to a cuvette and stoppered to reduce evaporation of the sample. The absorbance of the ODN was measured at 295 nm as the temperature of the sample was held for 10 min at 4°C, heated to 95°C at a rate of 0.5°C per min, then held at 95°C for 10 min before the process was reversed; each melting/annealing process was repeated three times. Data were recorded every 1°C during both melting and annealing and melting temperatures (*T*_m_) and annealing temperatures (*T*_a_) were determined using the first derivative method. This analysis was performed independently on each of the three melting curves collected for each ODN and the values presented are the average and standard deviation of these. TDS were calculated by subtracting the spectrum of the folded structure between 220 and 320 nm at 4°C from that of the unfolded structure at 95°C. The data was normalized and maximum change in absorption was set to +1 as previously described (28).

#### Circular dichroism

CD spectra were recorded on a Jasco J-810 spectropolarimeter using a 1 mm path length quartz cuvette. ODNs were diluted to 10 μM (total volume: 200 μl) in buffer at pH increments of 0.25 pH units from 5.0 to 8.0. The scans were recorded at room temperature between 200 and 320 nm. Data pitch was set to 0.5 nm and measurements were taken at a scanning speed of 200 nm/min, response time of 1 s, bandwidth of 2 nm and 100 mdeg sensitivity; each spectrum was the average of three scans. Samples containing only buffer were also scanned according to these parameters to allow for blank subtraction. Transitional pH (pH_T_) for each i-motif was calculated from the inflection point of fitted ellipticity at 288 nm.

#### Frontier orbital calculations

As previously described (29), model compounds were used in electronic structure calculations. Models of C, C^+^, C:G and C:C^+^ in single stranded, Watson–Crick paired duplex or i-motif structures in DNA, were 1-methyl-cytosine,1-methyl-N3-protonated-cytosine, 1-methly-cytosine Watson–Crick base paired with 1-methyl guanine, and 1–methyl-cytosine base paired with 1-methyl-N3-protonated-cytosine respectively. Electronic structure calculations for each of the above compounds and the HSO_3_^–^ anion were carried out in Spartan’18 (Wavefunction, Irvine, CA). Preliminary geometries obtained with molecular mechanics were submitted for equilibrium geometry calculation with Parametric Method 3. The resulting geometry was submitted for *ab initio* equilibrium geometry calculation using the Hartree–Fock level of theory with a final geometry optimization using the 6-31G* basis set. All molecular orbital parameters and surfaces were computed with this basis set.

#### Mass spectrometry analysis of (TCCC)_9_

Bisulfite mediated deamination of the (TCCC)_9_ oligodeoxynucleotide was carried out at 37°C and pH 5.3, where more than 95% of this oligodeoxynucleotide adopts an i-motif structure (see Table 2), using the procedure described above for non-denatured genomic DNA. Bisulfite treated DNA recovered from 0.5, 1 or 2 μg of input oligodeoxynucleotide was submitted to Novatia, LLC (Newtown, PA) for High Resolution Liquid Chromatography Mass Spectroscopic analysis. dC→dU conversions were detected as 1 Da increases in mass of the reference mass of the (TCCC)_9_ oligodeoxynucleotide. Deconvolution reports like the representative report given in Supplementary Data Set 1 were used to determine average frequencies for each level of conversion from 1 dU/36mer to 27 dU/36mer.

## RESULTS

### Bisulfite treatment of native genomic DNA

**Figure 1.**
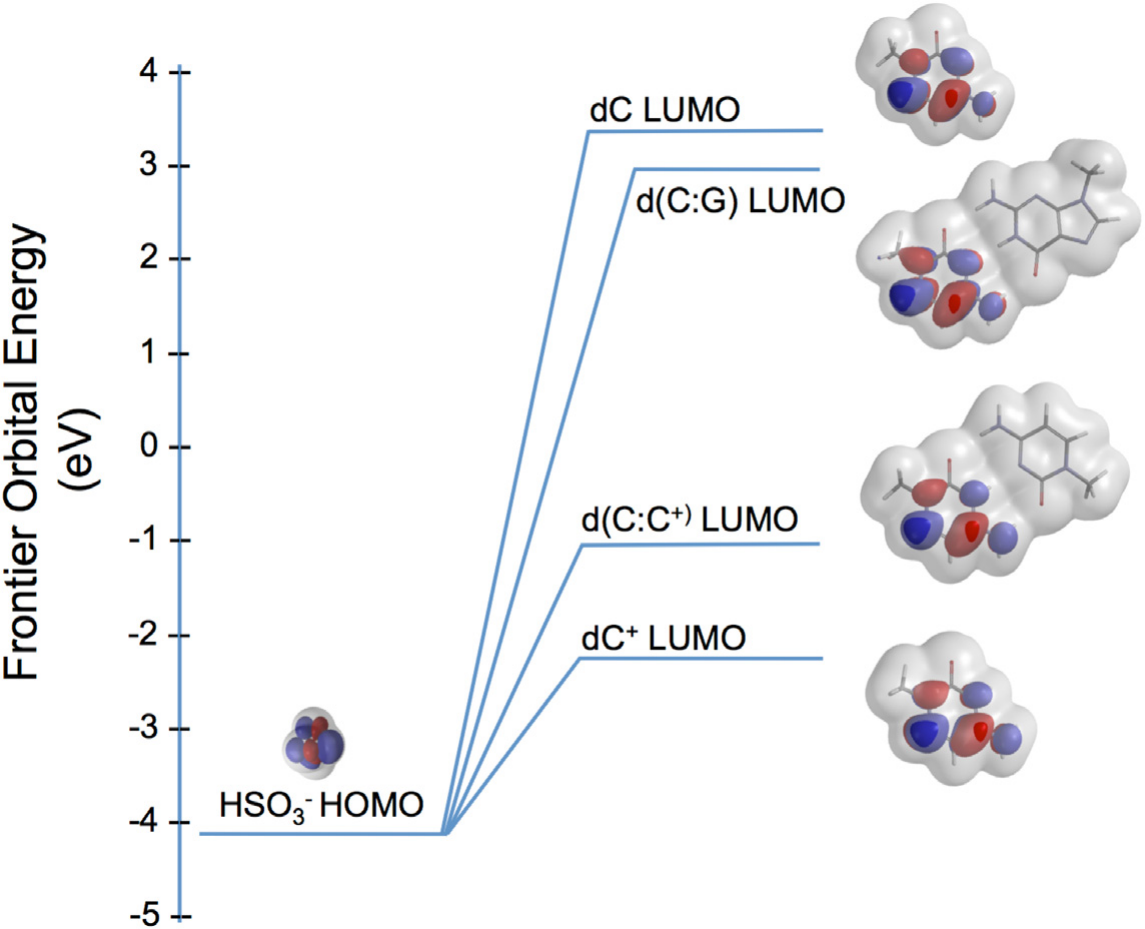
Frontier orbitals in the reaction between the bisulfite anion and cytosine in nucleic acids. The equilibrium geometry highest occupied molecular orbital (HOMO) for the bisulfite anion (lower left) is shown at its energy in eV under the transparent electron density that envelops of the molecule. Similar equilibrium geometry calculations, all at the Hartree-Fock 6–31G* level of theory, are depicted for the lowest unoccupied molecular orbital (LUMO) of models of cytosine in its several possible configurations in DNA. In each model the deoxyribose moiety at N1 of cytosine or guanine is modeled by a simple methyl group. Hence, the bases used in the electronic structure calculations are 1-methyl-cytosine, 1-methyl_cytoisne+ or 1-methyl-guanine. The geometry of the LUMO’s identify C6 of cytosine as the point of nucleophilic attack by the anion (blue center above the transparent electron density) in each model, and order the reactivity of each based on the LUMO energies in eV: dC^+^>d(C:C^+^)>>d(C:G)>dC.

At pH 5.3 and 37°C isolated DNA is primarily in the B-form DNA conformation with Watson-Crick base pairs. Biologically formed non-B structures can be expected to persist under these conditions, provided that they are not maintained by constraints present in chromatin (30,31). Cytosine residues that are Watson-Crick base paired in the B-DNA duplex are not susceptible to attack by the bisulfite nucleophile, which has been shown experimentally (31,32) and is confirmed by the frontier orbital energy of the C:G base pair model (Figure 1), where the lowest unoccupied molecular orbital (LUMO) of the base pair electrophile (centered on the cytosine moiety at C6) is very high relative to the highest occupied molecular orbital (HOMO) energy of the HSO_3_^−^ nucleophile. Consequently, for the regions comprising d(TCCC)_5_ and d(TCCC)_9_ depicted in Figure 3, bisulfite mediated deamination in native DNA at pH 5.3 and 37°C shows that these regions are in non-B structures on some chromosomes in PC3 cells. Deletions were also detected at the 5′ end of the d(TCCC)_5_ and d(TCCC)_9_ elements. Moreover, deamination and deletion at d(TCCC)_9_ was more extensive than it was at d(TCCC)_5_. Only two forms capable of reaction with the bisulfite nucleophile are possible at these sites: an unpaired and disordered loop, or an i-motif. Since pH 5.3 is near the p*K*_a_ of N3 of cytosine, should dissociation of a biologically formed i-motif occur during DNA isolation result in an unpaired loop, both the protonated and unprotonated forms of deoxycytosine would be present. While the frontier orbital picture virtually precludes nucleophilic attack by the HSO_3_^−^ anion on dC, given the high energy of its LUMO, the protonated dC^+^ residue is expected to be readily attacked as shown both by experiment (32,33) and by the proximity to its LUMO energy relative to the energy of the HOMO of HSO_3_^−^ (Figure 1).

**Figure 2.**
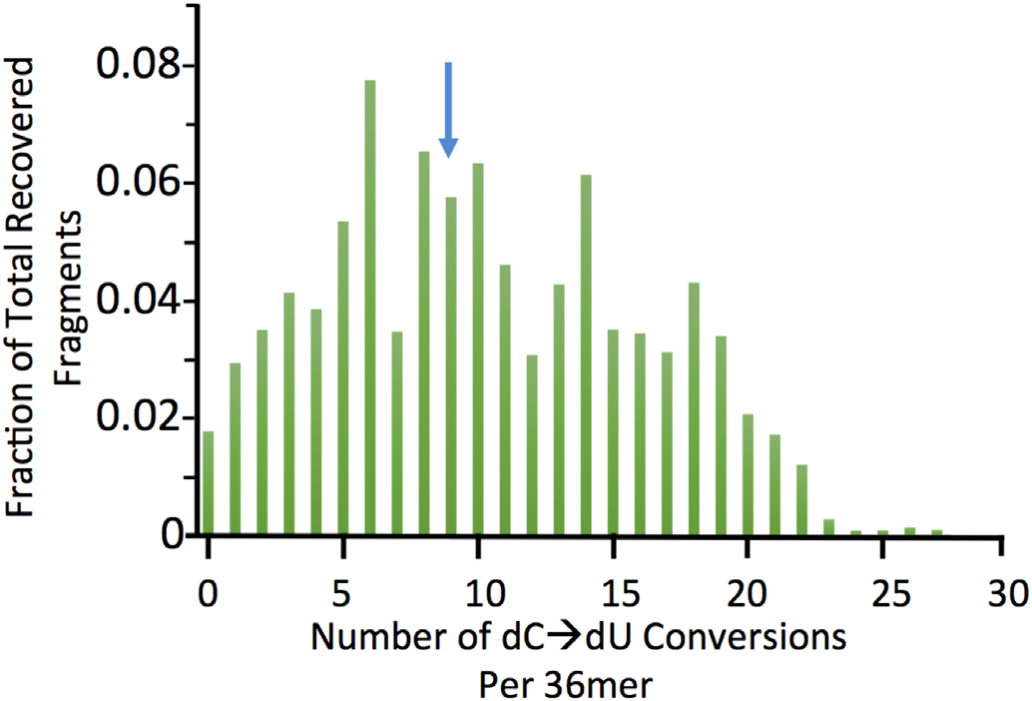
Frequency distribution for recovered 36mers after bisulfite treatment at 37°C and pH 5.3. Bisulfite treated DNA at 0.5, 1 and 2 μg input oligodeoxynucleotide DNA was recovered after high resolution liquid chromatography. Mass spectrometry showed that it is partially converted with a range of dU/36mer deaminations that are distributed from 0 to 27 dU deaminations in individual 36mers distinguished based on mass. The frequency of each species is plotted versus the number of dUs present in each 36mer as the average for the three input levels. The Distribution is centered on approximately 9dU/36mer (blue arrow). 92% of fragments in the spectrum had masses ≥ that of the d(TCCC)_9_ 36mer.

Although one might expect that an intact i-motif structure might resist bisulfite attack given the results with duplex DNA, the energy of the LUMO of the d(C:C^+^) base pair electrophile (centered over C6 of the dC^+^ moiety) in the i-motif (Figure 1) is also close to that of the nucleophile and therefore should be more readily attacked than either unprotonated dC or the C:G base pair. These considerations and the frontier orbital analysis above suggested that although the i-motif is a closed structure, it can be attacked by bisulfite. To directly confirm this possibility we treated d(TCCC)_9_ with bisulfite using the same procedure used for genomic DNA (i.e. pH 5.3 and 37°C) where it is almost exclusively in the i-motif form (Table 2). High resolution liquid chromatography mass spectrometry analysis showed that significant conversion of dC to dU occurred during the bisulfite reaction even though the i-motif structure was the dominant form in solution with a *T*_m_ of 66.7°C (Table 2). In these experiments intact 36mers accounted for 92% of the deconvoluted mass spectrum. A representative deconvolution report is given as Supplementary Data set 1. While the data clearly show significant conversion ranging from 1/27 to 27/27 cytosines converted. The frequency distribution for the recovered species was approximately random and peaks at about nine dC→dU conversions per 36mer (Figure 2). Unfortunately, sequence assignments are not possible with this method.

**Figure 3.**
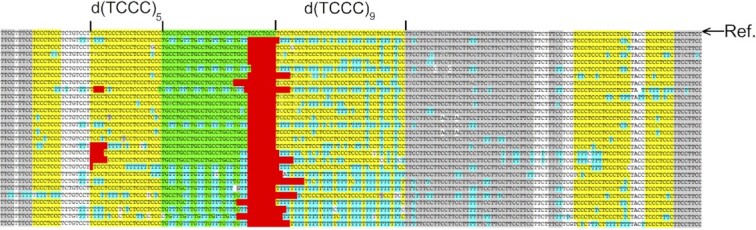
Bisulfite mediated deamination of native DNA in the region of RACK7 containing d(TCCC)_5_ and d(TCCC)_9_. After bisulfite treatment of native DNA at pH 5.3 and 37°C, representative cloned isolates of the region were sequenced and the sequences aligned under the genomic reference sequence from the GRCh38.p12 primary assembly of the human genome (marked Ref. in the figure). d(TCCC)*_n_* elements are depicted in yellow. The alignment shows that deletions of various length have occurred in different cells at the 5′ends of both d(TCCC)_5_ and d(TCCC)_9_. However, both the level of deamination (blue) and the level of deletion (red) are much more extensive at d(TCCC)_9_ than in d(TCCC)_5_.

The sequencing results given in Figure 3 show aligned exemplars originating from individual chromosomes. In spite of the deletions observed in many of the sequences, both the d(TCCC)_5_ and d(TCCC)_9_ regions are generally complete. The C→T conversion frequency in d(TCCC)_5_ was 10.4% while the conversion frequency in d(TCCC)_9_ is 37.4%. Further, deletions were confined to the 5′- end at both d(TCCC)_5_ and d(TCCC)_9_ with a frequency of 18.5% and 100% of the cloned sequences respectively.

### *In vitro* analysis of d(TCCC)_*n*_ and d(GGGA)_*n*_ model oligodeoxynucleotides

Given that the observed bisulfite mediated deamination was more extensive at d(TCCC)_9_ than at d(TCCC)_5_ we asked if the potential for i-motif structure formation at neutral pH was a function of length as seen in other i-motif forming sequences (22). Circular dichroism (CD) spectra exhibit well-characterized and easily distinguishable signatures for both the i-motif structure and the G-quadruplex structure for single-stranded oligodeoxynucleotide sequences. G-quadruplex sequences form spontaneously at neutral pH, however, i-motif structure formation is enhanced at low pH because protonation of N3 of cytosine increases the formation of the C:C^+^ base pair in parallel DNA strands. However, many i-motif structures are known to form effectively at neutral pH (4), suggesting that successive stacking interactions in longer i-motif forming sequences may also enhance i-motif formation.

We determined titration profiles for i-motif formation using CD spectra at different pHs for the d(TCCC)_*n*_ element with n ranging from 4 to 15. Data for the full analyses for the length series d(TCCC)_4_ to d(TCCC)_15_ is given in the Supplementary Figure S7A–I, and is summarized in Table 1. The input concentration of 10 μM oligodeoxynucleotide was chosen to permit detection at a concentration that favors unimolecular folding. The pH_T_ (i.e. the transition pH values at which equal concentration of i-motif and single strands are present in solution) for each sequence was seen to increase with increasing length. The pH_T_ values steadily increase with length up to a plateau with pH_T_ reaching neutral pH at lengths greater than n = 8 with corresponding average *T*_m_ values of 41°C (Table 1 and Supplementary Figure S8). In contrast, the equivalent G-rich sequences formed stable G-quadruplexes over the whole length range. When we performed analyses at physiological mimicking potassium cation concentrations (100 mM KCl), the *T*_m_ values were all >90°C, or near the limit of detection. Reduction of the KCl levels to 20 mM still gave average melting temperatures of >70°C for every sequence (Table 1 and Supplementary Figures S5A–H and Supplementary Table S1). This gives us confidence that in physiological conditions, these G-quadruplexes are highly stable, regardless of sequence length. Thermal difference spectra for G-quadruplex formation are given in Supplementary Figure S6.

**Table 1.** UV melting and annealing temperatures of d(TCCC) repeats determined by the first derivative method. DNA at 2.5 μM in 10 mM sodium cacodylate with 100 mM KCl at pH 6.5. Values are the mean from triplicate experiments with standard deviations. Transitional pH values determined by CD, with standard errors from the curve fit*

Sequence*	*T*_m_ (°C)	*T*_a_ (°C)	Hysteresis (°C)	pH_T_
**d(TCCC)_4_**	15.6 ± 1.0	13.8 ± 1.2	1.8 ± 2.1	6.6 ± 0.01
**d(TCCC)_5_**	28.3 ± 0.6	26.0 ± 0.6	2.4 ± 1.0	6.7 ± 0.04
**d(TCCC)_6_**	31.4 ± 0.6	29.2 ± 0.0	2.2 ± 0.6	6.8 ± 0.02
**d(TCCC)_7_**	32.8 ± 1.0	29.1 ± 0.0	3.7 ± 1.0	6.8 ± 0.03
**d(TCCC)_8_**	40.7 ± 0.6	30.7 ± 0.6	10.0 ± 0.0	6.9 ± 0.01
**d(TCCC)_9_**	40.7 ± 0.6	30.3 ± 0.6	10.4 ± 0.6	7.1 ± 0.04
**d(TCCC)_12_**	40.2 ± 0.6	30.5 ± 0.0	9.8 ± 0.6	7.2 ± 0.08
**d(TCCC)_14_**	42.0 ± 4.0	30.4 ± 0.0	11.6 ± 4.0	7.1 ± 0.07
**d(TCCC)_15_**	40.2 ± 4.0	23.2 ± 2.0	16.0 ± 2.0	7.1 ± 0.08

**Figure 4.**
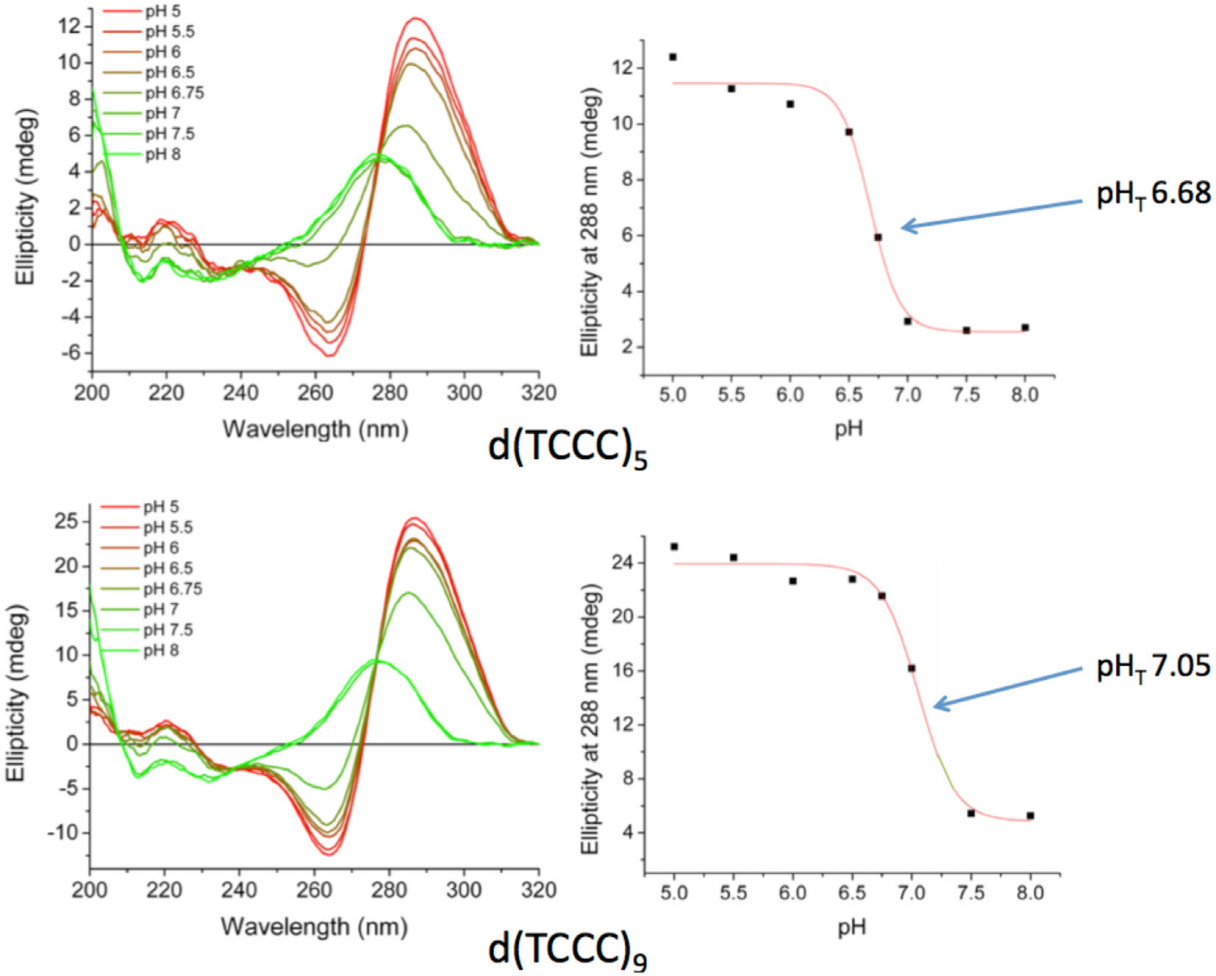
Titration curves for oligodeoxynucleotides with d(TCCC)_5_ and d(TCCC)_9_ repeats. CD spectra for each repeat over the titration range pH 5 to pH 8. In each titration, the characteristics i-motif signature emerge as pH is decreased, with the full signature emerging at pH 5 for d(TCCC)_5_ and at pH 5.5 for d(TCCC)_9_. Transition pH (pH_T_) values show that a significant fraction of each sequence would be present as i-motif near physiological pH.

The pH profiles for d(TCCC)_5_ and d(TCCC)_9_ are depicted in Figure 4. Both sequences have significant potential for the formation of i-motif structures at physiological pH, with the pH_T_ values of 6.7 for d(TCCC)_5_ and 7.1 for d(TCCC)_9_. Since the pH of the bisulfite reaction is very close to the p*K*_a_ of the N3 of cytosine, protonated cytosines are expected to be present during the reaction should a biologically formed i-motif dissociate into a loop. However, the CD titration profiles of each sequence suggest that at pH 5.3 a biologically formed i-motif would be preserved as the dominant form in solution during the bisulfite reaction and hence the primary substrate for the deamination reaction (Figure 4). Further, we assessed the stability of each of these sequences (d(TCCC)_5_ and d(TCCC)_9_) at pH 5.3 and, as expected, the melting temperatures were high (Supplementary Figure S4A and B). *T*_m_ profiles were determined for each d(TCCC)_n_ oligodeoxynucleotide at pH 6.7 and are given in Supplementary Figure S2A–I. Thermal difference spectra for i-motif formation are given in Supplementary Figure S3. At pH 6.5, the *T*_m_ value for d(TCCC)_5_ was found to be 28.3 ± 0.6°C and that for d(TCCC)_9_ was 40.7 ± 0.6°C (Table 1). However, at pH 5.3 the *T*_m_ value for d(TCCC)_5_ was found to be 50.2 ± 3.5°C and that for d(TCCC)_9_ was 66.7 ± 2.0°C (Table 2). This indicates that, under the conditions of the bisulfite reaction (pH 5.3 and 37°C), both these two regions would remain almost exclusively in the i-motif conformation in those chromosomes that had formed an i-motif biologically.

**Table 2.** UV melting and annealing temperatures of d(TCCC) repeats determined by the first derivative method. DNA at 2.5 μM in 10 mM sodium cacodylate with 100 mM KCl at pH 5.3. Values are the mean from triplicate experiments with standard deviations

Sequence**	*T*_m_ (°C)	*T*_a_ (°C)	Hysteresis (°C)
**d(TCCC)_5_**	50.2 ± 3.5	45.8 ± 3.5	4.3 ± 0.5
**d(TCCC)_9_**	66.7 ± 2.0	62.0 ± 2.0	4.6 ± 0.5

### Deletion Frequencies in the PC3 cell line

**Figure 5.**
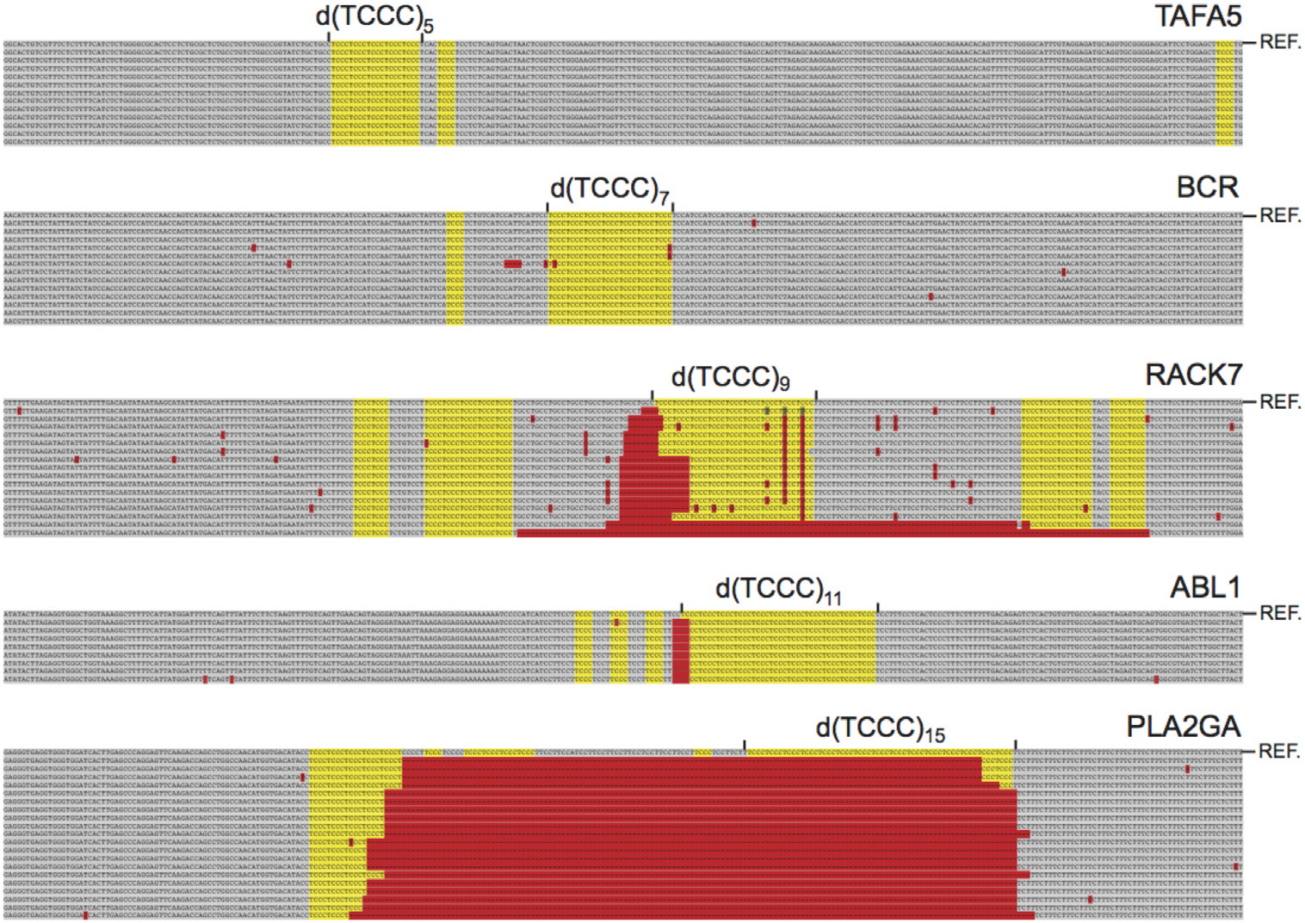
Deletions at multiple genomic locations in cultured human prostate cancer cells. Deleted sequences (red) are located near the 5′- end of the C-rich strand of the d(TCCC)_*n*_ repeat and often extend into the repeat itself. Deletions were not observed for d(TCCC)_5_ in the HAFA5 gene, and were very infrequent for d(TCCC)_7_ in the BCR gene, however for the d(TCCC)_9_ in the RACK7 gene, d(TCCC)_11_ in the ABL1 gene and d(TCCC)_15_ in the PLA2GA2 gene each cloned representative carried a deletion.

**Figure 6.**
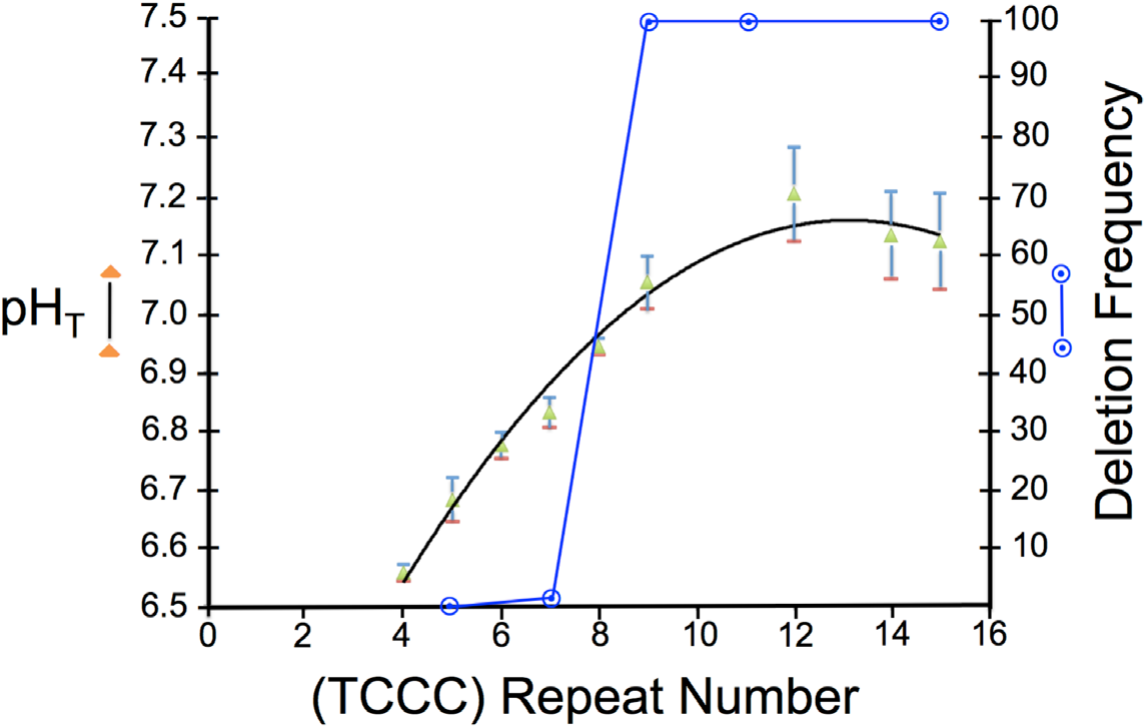
Length dependence of i-motif formation at neutral pH correlated with deletion frequency. pH_T_ value: closed triangles (green) ± standard deviation determined from the titration curve fit. Fraction of cloned sequences with a deletion: open circles (blue).

The d(TCCC)_*n*_ repetitive element occurs in multiple locations across the whole human genome. Of these, there are about 220 distinct locations were d(TCCC)_*n*_ for *n* ≥ 9. We used PCR to isolate fragments spanning sequences of various lengths from the genomic DNA of the PC3 cell line as previously described (26). Isolated fragments were cloned and sequenced also as described previously (26). Data in Figure 5 shows the results obtained for the HAFA5 gene (d(TCCC)_5_ element), the BCR gene (d(TCCC)_7_ element), the RACK7 gene (d(TCCC)_9_ element), the ABL1 gene (d(TCCC)_11_ element), and the PLA2G2A gene (d(TCCC)_15_ element). The sequences were aligned as previously described against the genomic sequence (GRCh38.p12 Primary Assembly) of the human genome (26). As depicted in Figure 5, deletions (highlighted in red) are uniformly associated with the 5′-end of the genomic sequence of the C-rich strand. Deletions are absent from cloned representatives of the HAFA5 site, with a single deletion recovered at the BCR site. However, the RACK7, ABL1 and PLA2G2A showed significant deletions at each cloned representative. The data is summarized in Figure 6. The potential formation of G-quadruplex structures is not correlated with deletion frequency at all, since in the absence of an i-motif (i.e. as a single-stranded oligodeoxynucleotide), G-quadruplex structures were observed to form *in vitro* at physiological pH at each length tested (Supplementary Figure S9). Moreover, even at sub-physiological potassium levels (20 mM KCl) all the melting temperatures for the G-quadruplex forming sequences were above 70°C (see Supplementary Figure S5A–H and Supplementary Table S1). Thermal difference spectra for G-quadruplex formation are given in Supplementary Figure S6. Conversely, we observe a strong correlation between the potential for i-motif formation *in vitro* and the frequency of deletion in PC3, since 100% of the recovered clones showed a deletion when the pH_T_ of the relevant i-motif forming sequence observed *in vitro* is above pH 7.0.

## DISCUSSION

While G-quadruplex and i-motif structures can both form at neutral pH, as noted here for the d(TCCC)_n_ and as reported for dC_n_ (22), i-motif structure formation at neutral pH can depend dramatically on sequence length and composition. Consequently, the correlation between deletion frequency and d(TCCC)_n_ folding at neutral pH (Figure 6) provides strong evidence that i-motif structures are linked to deletions at d(TCCC)_n_ elements in the human genome. This correlation suggests that i-motif structures form *in vivo* in the repair compromised PC3 cell line. Further, the *in vitro* findings of pH_T_’s near 7.1 and a melting temperatures above 40°C, for each sequence length associated with a significant deletion frequency in PC3 (Supplementary Figures S2A-I, S7A-I and S8), indicate that the i-motif structure, once formed, is expected maintain a structural disruption of duplex DNA structure at 37°C and physiological pH at d(TCCC)_n_ with n ≥ 9.

Even so, we can only speculate on the process that leads to the extrusion of G-quadruplexes and i-motifs biologically. Evidence suggesting roles in transcription require complex biological processes that elicit these structures (7,8,11,34). Such systems might alter local topology in order to extrude quadruplexes and it is known that transcription itself can extrude a G-quadruplex at a site of RNA:DNA hybrids during R-Loop formation (35). However, i-motif structures have not been found associated with R-Loops, since RNA:DNA hybrids appear to form exclusively on the C-rich strand in regions of GC-Skew.

That one or both forms are in fact extruded so as to elicit replication stress is strongly suggested by the data in *C. elegans* (20,21,36) and our own data on the human PC3 cell line (26). In *C. elegans* dog-1 mutant worms, unresolved G-quadruplex structures cause replication stress and replication fork stalling and collapse (37). Since replication fork stalling can lead to HMG helicase uncoupling (38), once a replication fork stalls and the HMG helicase uncouples (38) the two strands will be separated and both strands can form non-B structures as topological constraints outside the region are relieved. In previous work we used a cloning procedure (see Supplementary Figure 1) designed to clone sequences near uncoupled HMG halt sites (26). Clones isolated from PC3 cell line DNA using that procedure were most often within 5 kb of a G-quadruplex motif (26). The d(TCCC)_9_ site in RACK7 was identified with this cloning procedure suggesting that the clone came from a site undergoing replication stress and HMG helicase uncoupling (26).

Our previous work on the RACK7 repeat in PC3 cells also showed that d(TCCC)_9_ sequence at this site was heavily deaminated by bisulfite under non-denaturing conditions (26), demonstrating that it is not paired with its complementary strand (31). However, the data presented here suggests that the i-motif structure can even survive after DNA isolation, as has been shown for other non-B structures (30,31). Importantly, the frequency of deamination at the d(TCCC)_5_ element in the region of 10% compared to the frequency of 37% for d(TCCC)_9_ correlates with the relative lower stability of d(TCCC)_5_ (pH_T_ 6.7 and *T*_m_ = 28°C) compared to d(TCCC)_9_ (pH_T_ 7.1 and *T*_m_ = 41°C) under physiological conditions. We considered the possibility that the region around d(TCCC)_9_ was simply single-stranded and the i-motif might not be present given the possibility that, if it were present, it might behave like native duplex DNA and would resist nucleophilic attack by bisulfite. However, careful analysis of the features of the bisulfite reaction (Figure 1) and pH and melting profiles (Table 2 and Figure 4) suggested that: the i-motif itself is the mostly likely initial substrate for nucleophilic attack by the bisulfite anion at pH 5.3 and 37°C (Figure 3). This suggestion was confirmed by high resolution liquid chromatography mass spectrometry of the bisulfite treated d(TCCC)_9_ 36mer (Figure 2) at 37°C and pH 5.3. After bisulfite treatment, between 1/27→27/27 dC→dU conversions were observed in intact 36mers in a roughly random distribution (Figure 2). While the method does not allow sequence assignments for the conversions, the approximate peak in the frequency distribution (Figure 2) at 9 conversions/ 36mer is consistent with the frequency of 10.8 conversions/36mer seen in bisulfite treated genomic DNA (Figure 3).

Further, the roughly random distribution of frequencies is consistent with the frontier orbital picture (Figure 1) since in the initial stages of the reaction, cytosines exposed in a loop would be expected to be only marginally more reactive than those in a closed i-motif. As the reaction progressed, bisulfite addition to the i-motif structure is expected to destabilize the structure by neutralizing the charge on the protonated cytosine and introducing sp3 carbons at both C6 and C5. Such destabilization is expected to permit further bisulfite addition at exposed dC^+^ residues in unpaired single-strands.

**Figure 7.**
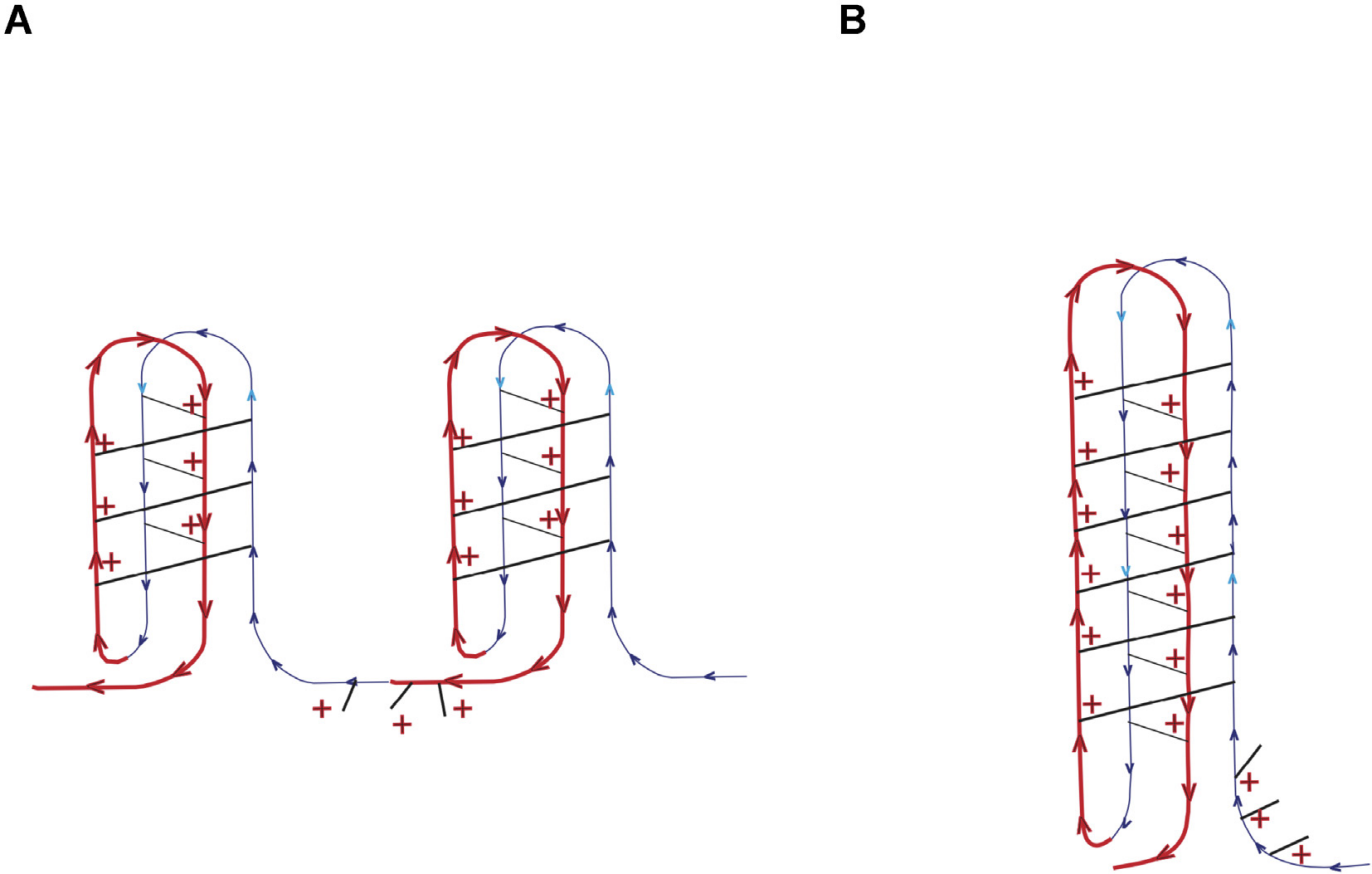
Possible structures for d(TCCC)_9_. (**A**) A Tandem Structure Composed of two linked i-motifs formed from two d(TCCC)_4_ elements linked by a single d(TCCC): d(TCCC)_4_–d(TCCC)–d(TCCC)_4_. (**B**) A single d(TCCC)_9_ i-motif. In each structure the loops contain a single dT residue.

While the mass spectrometry data does not address the nature of the structure of the i-motif that forms in d(TCCC)_9,_ for a sequence of this type with 27 dC residues, two forms seem plausible. In the first form the sequence can be viewed as two i-motif 16mers linked as follows: d(TCCC)_4_–d(TCCC)–d(TCCC)_4_ (Figure 7A). In the second a single 32mer forms the i-motif linked as follows: d(TCCC)–d(TCCC)_8_. Although our data do not prove that the structure is more like the second structure (Figure 7B) one expects the *T*_m_ for the tandem structure (Figure 7A) to be very close to that observed for d(TCCC)_4_ at neutral pH *i.e*. 15.6°C as opposed to the observed value for d(TCCC)_9_ at neutral pH of 40.7°C (Table 1A). In both models (Figure 7) a single dT residue is exposed in the loops of each i-motifs. Additional stability in a longer structure, like that depicted in Figure 7B, may be achieved in part by the additional stacking energy and in part by the increase in pKa associated with additional base pairing (39,40).

In addition to these findings it is important to note that independent of d(TCCC) repeat length, all of the deletions we observe, mapped near the 5′ end of C-rich strand of the d(TCCC)_*n*_ element and often extended into the element itself (Figure 3). The opposite orientation for deletions has been clearly shown for dog-1 mutations in *C. elegans* (20,21). There, deletions map 5′- to the G-rich strand and G-quadruplex provides the impetus for deletion. Hence, the orientation of the human deletions reported here implicates i-motif formation during or prior to DNA replication in the genesis of deletions at d(TCCC)_n_ sequences.

By analogy with the multiple repair systems known to play roles in suppressing G-quadruplex formation, it seems likely that similar suppression systems for i-motif formation may exist, given the association of the i-motif with deletions as described here. However, the efficiency of such systems remains unknown. In this regard, it must be noted that the PC3 cell line has been in long-term cell culture, hence the deletions we see may also indicate a simple stochastic accumulation of deletions representing the overall frequency of i-motif occurrence and the efficiency of suppression at each replication cycle. In this regard, it is interesting to note that if the *in vitro* pH_T_ values for d(TCCC)_*n*_ sequences for *n* ≥ 9 reflect a high rate of i-motif formation at these sites, and the overall efficiency of an i-motif suppression system translates to a probability of remaining unsuppressed *in vivo* during replication of only 0.05, then the probability that an i-motif would impair replication and induce DNA damage on average at least once in 25 replication cycles is 0.72.

**Figure 8.**
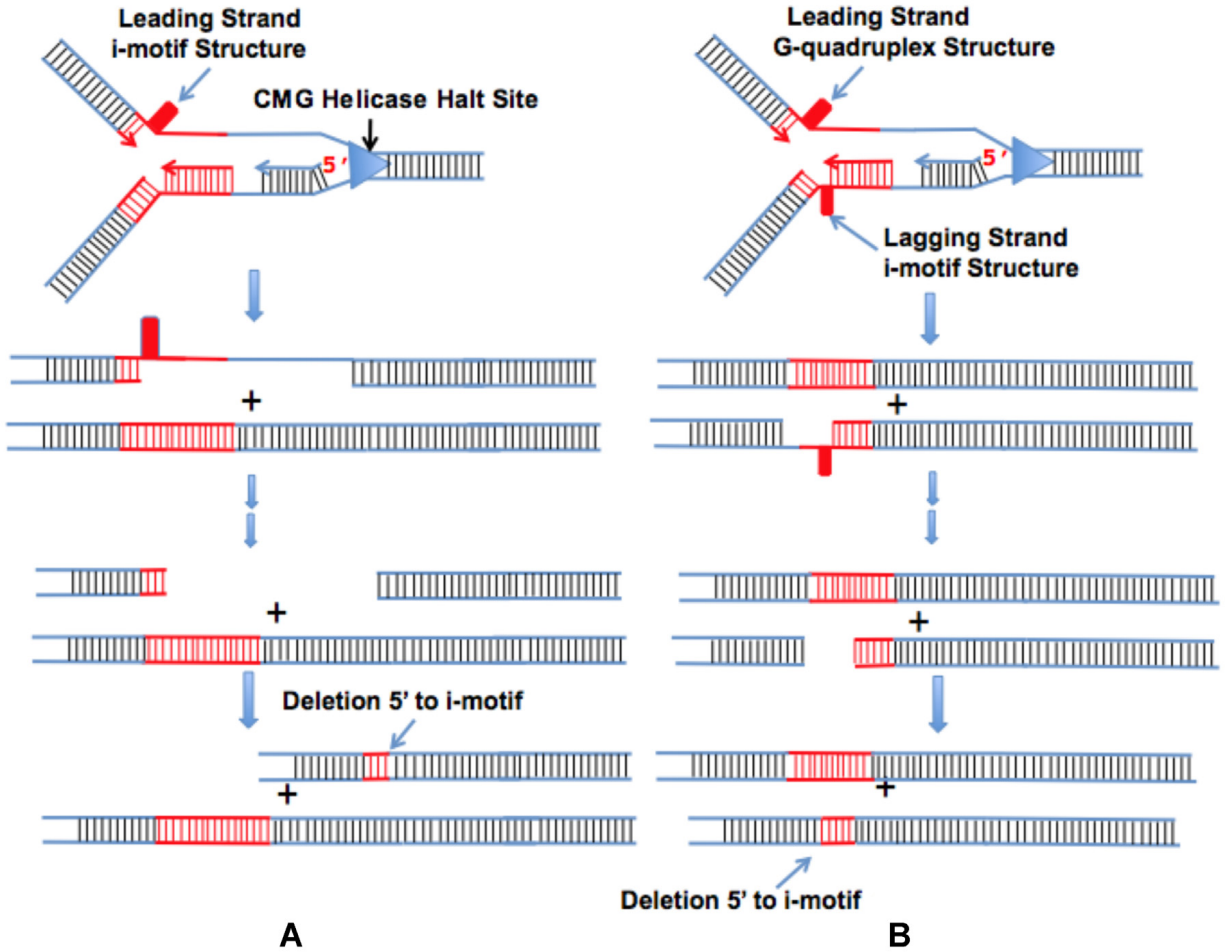
i-Motif induced deletion models. i-Motifs can form on either the leading or lagging strand depending on the direction of replication. When replication completes, either by restarting replication beyond the leading strand impediment or by replication from a distant replication origin two types of structure are created (20,21,36). A: When an i-motif forms the leading strand impediment and survives after replication deletions occur 5′ to the i-motif on the C-rich strand often extending into the repeat sequence itself. B: When a G-quadruplex forms a transient leading strand impediment and an i-motif forms on the lagging strand, deletions again occur near the i-motif often extending into the repeat sequence itself. Note that when only G-quadruplex survives replication as is the case in dog-1 mutant *C. elegans* deletions will occur 5′ to the G-quadruplex on the G-rich strand (20,21,36) often extending into the repeat itself.

Models of the deletion process induced by the *dog-1* mutation in *C. elegans* suggest that poorly suppressed G-quadruplexes provide a replication impediment that creates a gap beyond the impediment that survives replication and leads to deletions in a subsequent replication cycle that map to 5′ end of the impediment (21,37). Our findings in the human PC3 cell line for i-motif impediments formed at the d(TCCC)_*n*_ elements are completely consistent with this model (Figure 8). The data presented here and in (26) would support HMG helicase uncoupling and uncoupled replication followed by leading strand repriming to produce a gap (41) near an i-motif leading strand impediment (Figure 8A). A transient G-quadruplex that led to HMG helicase uncoupling and uncoupled replication and the formation of an i-motif on the lagging strand would leave a gap in the lagging strand 3′ to the i-motif (Figure 8B). Such a gap would explain why bisulfite can attack the region 3′ to d(TCCC)_9_ element in RACK7 (Figure 3). Given that the dog-1 mutation impairs G-quadruplex suppression and results in deletions mapping 5′ to end of the G-quadruplex in the dG_n_ elements in *C. elegans*, models like that shown in Figure 8A in which the leading strand impediment is an unresolved G-quadruplex are ruled out since they require that the deletions occur 5′ to the G-quadruplex impediment on the G-rich strand and 3′ on the C-rich strand, because all of the deletions we observe are 5′- to the i-motif/G-quadruplex forming sequence on the C-rich strand. Given the the ability of the d(GGGA)_n_ motifs to form G-quadruplexes *in vitro* over the range of lengths n > 3 we conclude that while G-quadruplex formation at these sites may be a necessary or contributing condition for these deletions, i-motif formation a under physiological conditions is required for deletions to occur.

Finally, given the dog-1 precedent indicating that loss of a G-quadruplex resolvase results in deletions at sites that can form G-quadruplexes and i-motifs it is reasonable to speculate that the failure of an unknown i-motif suppression system could result in the deletions in the deletions observed here in the PC3 cell line. Given the absence of a functional p53 gene in the PC3 cell line, failure to suppress i-motif formation may be linked to p53, however, direct evidence for this suggestion is currently unavailable.

## Supplementary Material

gkac158_Supplemental_Files

## References

[1] Huppert J.L. , Balasubramanian S. Prevalence of quadruplexes in the human genome. Nucleic Acids Res. 2005; 33:2908–2916. 15914667 10.1093/nar/gki609 PMC1140081

[2] Sundquist W.I. , Klug A. Telomeric DNA dimerizes by formation of guanine tetrads between hairpin loops. Nature. 1989; 342:825–829. 2601741 10.1038/342825a0

[3] Del Mundo I.M.A. , Zewail-Foote M., Kerwin S.M., Vasquez K.M. Alternative DNA structure formation in the mutagenic human c-MYC promoter. Nucleic Acids Res. 2017; 45:4929–4943. 28334873 10.1093/nar/gkx100 PMC5416782

[4] Day H.A. , Pavlou P., Waller Z.A. i-Motif DNA: structure, stability and targeting with ligands. Bioorg. Med. Chem. 2014; 22:4407–4418. 24957878 10.1016/j.bmc.2014.05.047

[5] Smith S.S. Evolutionary expansion of structurally complex DNA sequences. Cancer Genomics Proteomics. 2010; 7:207–215. 20656986

[6] Wu F. , Niu K., Cui Y., Li C., Lyu M., Ren Y., Chen Y., Deng H., Huang L., Zheng S. et al. Genome-wide analysis of DNA G-quadruplex motifs across 37 species provides insights into G4 evolution. Commun Biol. 2021; 4:98. 33483610 10.1038/s42003-020-01643-4 PMC7822830

[7] Huppert J.L. , Balasubramanian S. G-quadruplexes in promoters throughout the human genome. Nucleic Acids Res. 2007; 35:406–413. 17169996 10.1093/nar/gkl1057 PMC1802602

[8] Kendrick S. , Kang H.J., Alam M.P., Madathil M.M., Agrawal P., Gokhale V., Yang D., Hecht S.M., Hurley L.H. The dynamic character of the BCL2 promoter i-motif provides a mechanism for modulation of gene expression by compounds that bind selectively to the alternative DNA hairpin structure. J. Am. Chem. Soc. 2014; 136:4161–4171. 24559410 10.1021/ja410934b PMC3985915

[9] Clark J. , Smith S.S. Secondary structure at a hot spot for DNA methylation in DNA from human breast cancers. Cancer Genomics Proteomics. 2008; 5:241–251. 19129555 PMC2989428

[10] Sarkies P. , Reams C., Simpson L.J., Sale J.E. Epigenetic instability due to defective replication of structured DNA. Mol. Cell. 2010; 40:703–713. 21145480 10.1016/j.molcel.2010.11.009 PMC3145961

[11] Mao S.Q. , Ghanbarian A.T., Spiegel J., Martinez Cuesta S., Beraldi D., Di Antonio M., Marsico G., Hansel-Hertsch R., Tannahill D., Balasubramanian S. DNA G-quadruplex structures mold the DNA methylome. Nat. Struct. Mol. Biol. 2018; 25:951–957. 30275516 10.1038/s41594-018-0131-8 PMC6173298

[12] Smith S.S. Stalling of DNA methyltransferase in chromosome stability and chromosome remodelling (Review). Int. J. Mol. Med. 1998; 1:147–156. 9852213

[13] Ginno P.A. , Lott P.L., Christensen H.C., Korf I., Chedin F. R-loop formation is a distinctive characteristic of unmethylated human CpG island promoters. Mol. Cell. 2012; 45:814–825. 22387027 10.1016/j.molcel.2012.01.017 PMC3319272

[14] Sun H. , Karow J.K., Hickson I.D., Maizels N. The bloom's syndrome helicase unwinds G4 DNA. J. Biol. Chem. 1998; 273:27587–27592. 9765292 10.1074/jbc.273.42.27587

[15] Fry M. , Loeb L.A. Human werner syndrome DNA helicase unwinds tetrahelical structures of the fragile x syndrome repeat sequence d(CGG)n. J. Biol. Chem. 1999; 274:12797–12802. 10212265 10.1074/jbc.274.18.12797

[16] Wu Y. , Shin-ya K., Brosh R.M. Jr FANCJ helicase defective in fanconia anemia and breast cancer unwinds G-quadruplex DNA to defend genomic stability. Mol. Cell. Biol. 2008; 28:4116–4128. 18426915 10.1128/MCB.02210-07 PMC2423121

[17] Eddy S. , Ketkar A., Zafar M.K., Maddukuri L., Choi J.Y., Eoff R.L. Human rev1 polymerase disrupts G-quadruplex DNA. Nucleic Acids Res. 2014; 42:3272–3285. 24366879 10.1093/nar/gkt1314 PMC3950705

[18] Betous R. , Rey L., Wang G., Pillaire M.J., Puget N., Selves J., Biard D.S., Shin-ya K., Vasquez K.M., Cazaux C. et al. Role of TLS DNA polymerases eta and kappa in processing naturally occurring structured DNA in human cells. Mol. Carcinog. 2009; 48:369–378. 19117014 10.1002/mc.20509 PMC2696892

[19] Kononenko A.V. , Ebersole T., Vasquez K.M., Mirkin S.M. Mechanisms of genetic instability caused by (CGG)n repeats in an experimental mammalian system. Nat. Struct. Mol. Biol. 2018; 25:669–676. 30061600 10.1038/s41594-018-0094-9 PMC6082162

[20] Kruisselbrink E. , Guryev V., Brouwer K., Pontier D.B., Cuppen E., Tijsterman M. Mutagenic capacity of endogenous G4 DNA underlies genome instability in FANCJ-defective c. elegans. Curr. Biol. 2008; 18:900–905. 18538569 10.1016/j.cub.2008.05.013

[21] Koole W. , van Schendel R., Karambelas A.E., van Heteren J.T., Okihara K.L., Tijsterman M. A polymerase Theta-dependent repair pathway suppresses extensive genomic instability at endogenous G4 DNA sites. Nat. Commun. 2014; 5:3216. 24496117 10.1038/ncomms4216

[22] Fleming A.M. , Ding Y., Rogers R.A., Zhu J., Zhu J., Burton A.D., Carlisle C.B., Burrows C.J. 4n-1 is a "Sweet spot" in DNA i-Motif folding of 2′-Deoxycytidine homopolymers. Nat. Commun. 2017; 139:4682–4689. 10.1021/jacs.6b10117 28290680

[23] Cui Y. , Kong D., Ghimire C., Xu C., Mao H. Mutually exclusive formation of G-Quadruplex and i-Motif is a general phenomenon governed by steric hindrance in duplex DNA. Biochemistry. 2016; 55:2291–2299. 27027664 10.1021/acs.biochem.6b00016

[24] King J.J. , Irving K.L., Evans C.W., Chikhale R.V., Becker R., Morris C.J., Pena Martinez C.D., Schofield P., Christ D., Hurley L.H. et al. DNA G-quadruplex and i-Motif structure formation is interdependent in human cells. J. Am. Chem. Soc. 2020; 142:20600–20604. 33253551 10.1021/jacs.0c11708

[25] Wolski P. , Nieszporek K., Panczyk T. G-Quadruplex and I-Motif structures within the telomeric DNA duplex. A molecular dynamics analysis of protonation states as factors affecting their stability. J. Phys. Chem. B. 2019; 123:468–479. 30589547 10.1021/acs.jpcb.8b11547

[26] Amparo C. , Clark J., Bedell V., Murata-Collins J.L., Martella M., Pichiorri F., Warner E.F., Abdelhamid M.A.S., Waller Z.A.E., Smith S.S. Duplex DNA from sites of helicase-polymerase uncoupling links Non-B DNA structure formation to replicative stress. Cancer Genomics Proteomics. 2020; 17:101–115. 32108033 10.21873/cgp.20171 PMC7078840

[27] Munson K. , Clark J., Lamparska-Kupsik K., Smith S.S. Recovery of bisulfite-converted genomic sequences in the methylation-sensitive QPCR. Nucleic Acids Res. 2007; 35:2893–2903. 17439964 10.1093/nar/gkm055 PMC1888819

[28] Mergny J.L. , Li J., Lacroix L., Amrane S., Chaires J.B. Thermal difference spectra: a specific signature for nucleic acid structures. Nucleic. Acids. Res. 2005; 33:e138. 16157860 10.1093/nar/gni134 PMC1201377

[29] Clark J. , Shevchuk T., Kho M.R., Smith S.S. Methods for the design and analysis of oligodeoxynucleotide-based DNA (cytosine-5) methyltransferase inhibitors. Anal. Biochem. 2003; 321:50–64. 12963055 10.1016/s0003-2697(03)00402-0

[30] Raghavan S.C. , Swanson P.C., Wu X., Hsieh C.L., Lieber M.R. A non-B-DNA structure at the bcl-2 major breakpoint region is cleaved by the RAG complex. Nature. 2004; 428:88–93. 14999286 10.1038/nature02355

[31] Raghavan S.C. , Tsai A., Hsieh C.L., Lieber M.R. Analysis of non-B DNA structure at chromosomal sites in the mammalian genome. Methods Enzymol. 2006; 409:301–316. 16793408 10.1016/S0076-6879(05)09017-8

[32] Shapiro R. , Braverman B., Louis J.B., Servis R.E. Nucleic acid reactivity and conformation. II. Reaction of cytosine and uracil with sodium bisulfite. J. Biol. Chem. 1973; 248:4060–4064. 4736082

[33] Hayatsu H. , Wataya Y., Kazushige K. The addition of sodium bisulfite to uracil and to cytosine. J. Am. Chem. Soc. 1970; 92:724–726. 5411063 10.1021/ja00706a062

[34] Guo K. , Gokhale V., Hurley L.H., Sun D Intramolecularly folded G-quadruplex and i-motif structures in the proximal promoter of the vascular endothelial growth factor gene. Nucleic Acids Res. 2008; 36:4598–4608. 18614607 10.1093/nar/gkn380 PMC2504309

[35] Duquette M.L. , Pham P., Goodman M.F., Maizels N. AID binds to transcription-induced structures in c-MYC that map to regions associated with translocation and hypermutation. Oncogene. 2005; 24:5791–5798. 15940261 10.1038/sj.onc.1208746

[36] Lemmens B. , van Schendel R., Tijsterman M. Mutagenic consequences of a single G-quadruplex demonstrate mitotic inheritance of DNA replication fork barriers. Nat. Commun. 2015; 6:8909. 26563448 10.1038/ncomms9909 PMC4654259

[37] van Kregten M. , Tijsterman M. The repair of G-quadruplex-induced DNA damage. Exp. Cell. Res. 2014; 15:178–183. 10.1016/j.yexcr.2014.08.038 25193076

[38] Taylor M.R.G. , Yeeles J.T.P. Dynamics of replication fork progression following helicase-polymerase uncoupling in eukaryotes. J. Mol. Biol. 2019; 431:2040–2049. 30894292 10.1016/j.jmb.2019.03.011 PMC6525111

[39] Wright E.P. , Huppert J.L., Waller Z.A.E. Identification of multiple genomic DNA sequences which form i-motif structures at neutral pH. Nucleic Acids Res. 2017; 45:13095–13096. 28180276 10.1093/nar/gkx090 PMC5605235

[40] Skolakova P. , Renciuk D., Palacky J., Krafcik D., Dvorakova Z., Kejnovska I., Bednarova K., Vorlickova M. Systematic investigation of sequence requirements for DNA i-motif formation. Nucleic Acids Res. 2019; 47:2177–2189. 30715498 10.1093/nar/gkz046 PMC6412112

[41] Yeeles J.T.P. , Poli J., Marians K.J., Pasero P. Rescuing stalled or damaged replication forks. Cold Spring Harb. Perspect. Biol. 2013; 5:a012815. 23637285 10.1101/cshperspect.a012815 PMC3632063

