# Supplementary material for: i-Motif formation and spontaneous deletions in human cells
Source: Nucleic Acids Res. 2022 Mar 7;50(6):3445–55. doi: 10.1093/nar/gkac158 (PMC8989526; doi:10.1093/nar/gkac158)
Supplement: gkac158_Supplemental_Files [file gkac158_supplemental_files.zip › 18Jan22FNL_Supp_InfoACC_ALL.pdf]

Supplementary Information

i-Motif Formation and Spontaneous Deletions in Human Cells

Marianna Martella, Flavia Pichiorri, Rupesh V. Chikhale, Mahmoud A. S. Abdelhamid , Zoë A. E. Waller, and Steven S. Smith

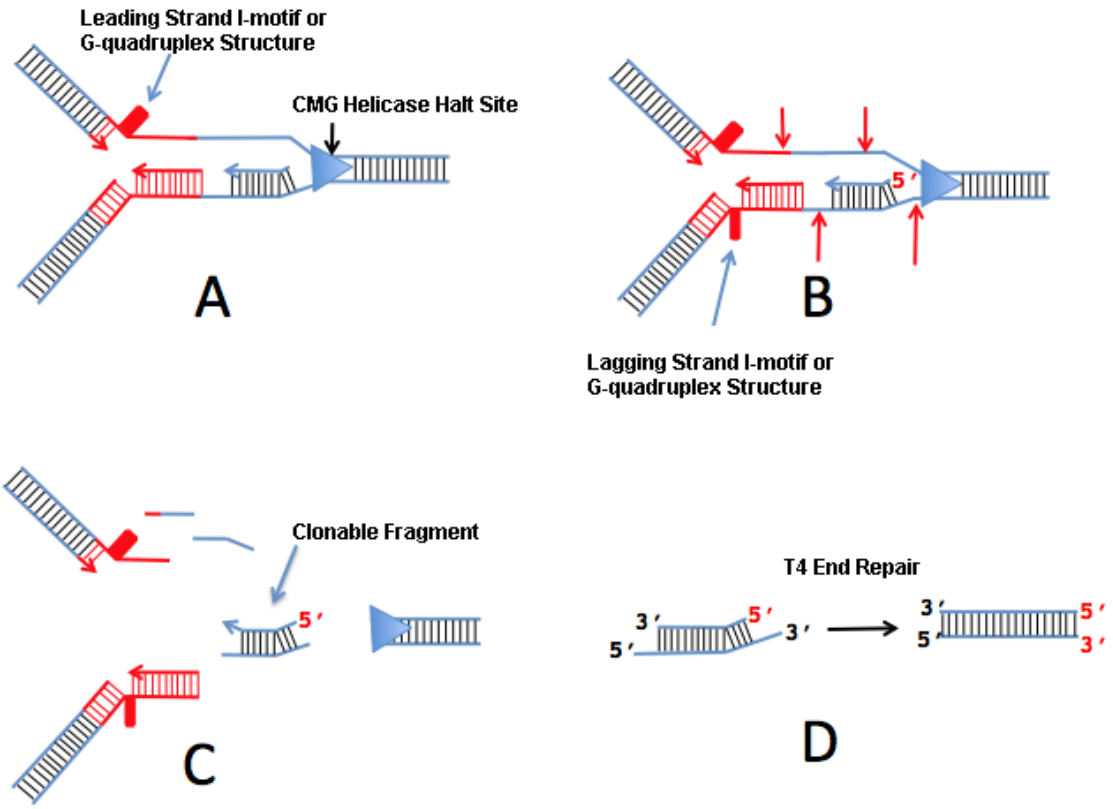

Figure S1: In Previous work (26) we cloned double stranded fragments from freshly isolated DNA on the assumption these sequences would sometimes originate from stalled replication intermediates of the type shown in **A** where HMG helicase uncoupling has occurred. Random shear during DNA isolation was expected to occur a the red arrows shown in **B**, yielding a clonable fragment shown in **C**, that could be end repaired as shown in **D** and blunt end cloned for sequencing and chromosomal location determination. Sequences were recovered at exceptionally high frequency from the RACK7 gene. Each clone had a common break point which was interpreted as the break nearest the helicase halt site, and randomly placed breaks distal to a non-B structure forming sequence capable of both G-quadruplex and i-motif formation at neutral pH.

UV Absorption Spectroscopy on Length Variants

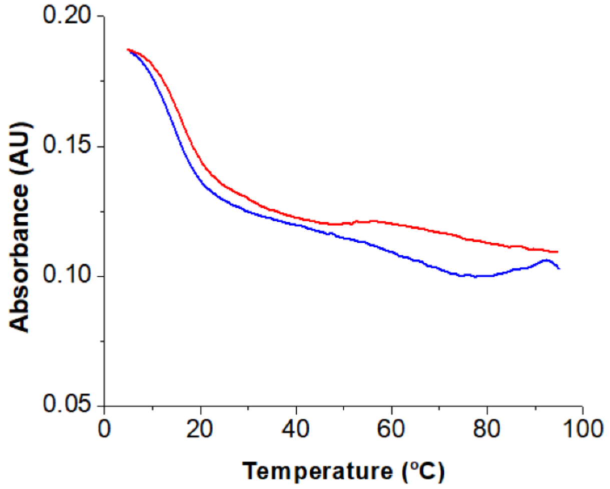

Figure S2A. Example UV melting (red) and annealing (blue) curves for d(TCCC)$_4$ at 2.5 µM in 10 mM sodium cacodylate with 100 mM KCl at pH 6.5.

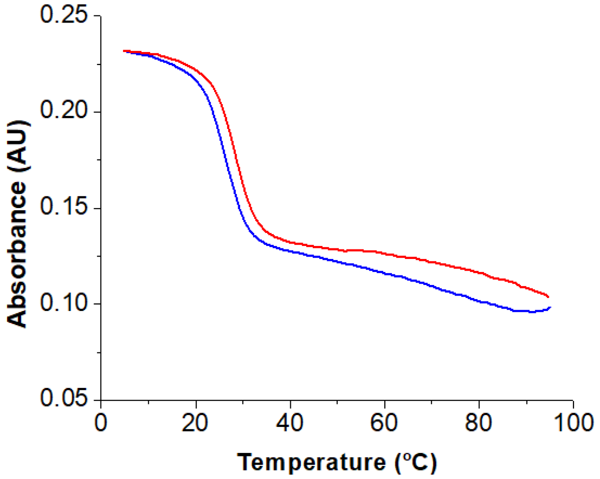

Figure S2B. Example UV melting (red) and annealing (blue) curves for d(TCCC)$_5$ at 2.5 µM in 10 mM sodium cacodylate with 100 mM KCl at pH 6.5.

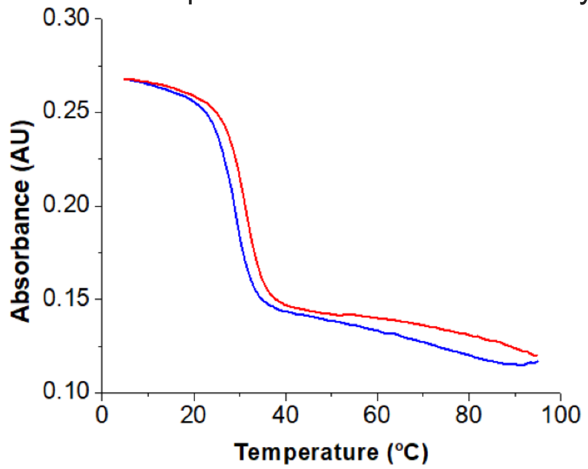

Figure S2C. Example UV melting (red) and annealing (blue) curves for d(TCCC)$_6$ at 2.5 μM in 10 mM sodium cacodylate with 100 mM KCl at pH 6.5.

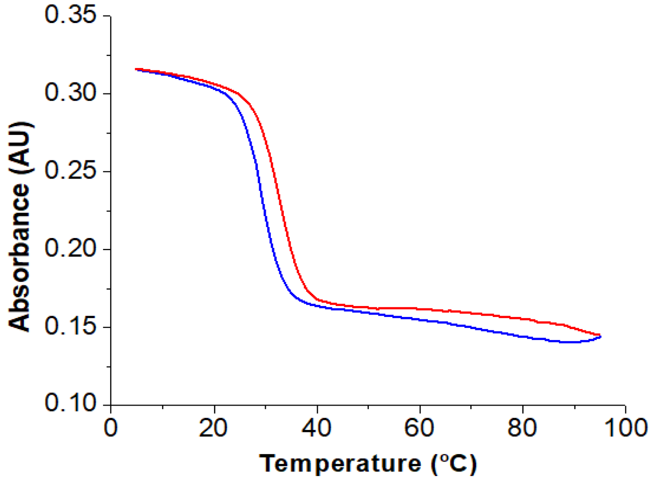

Figure S2D. Example UV melting (red) and annealing (blue) curves for d(TCCC)$_7$ at 2.5 μM in 10 mM sodium cacodylate with 100 mM KCl at pH 6.5.

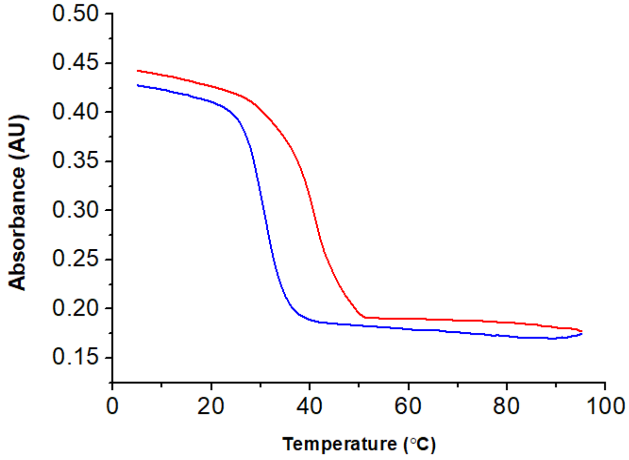

Figure S2E. Example UV melting (red) and annealing (blue) curves for d(TCCC)$_8$ at 2.5 μM in 10 mM sodium cacodylate with 100 mM KCl at pH 6.5.

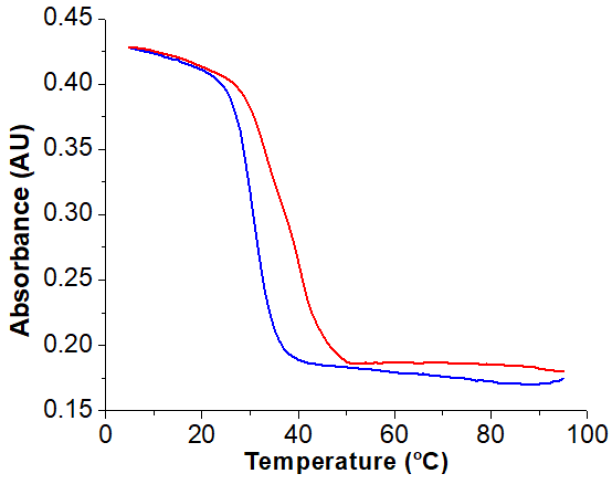

Figure S2F. Example UV melting (red) and annealing (blue) curves for d(TCCC)$_9$ at 2.5 μM in 10 mM sodium cacodylate with 100 mM KCl at pH 6.5.

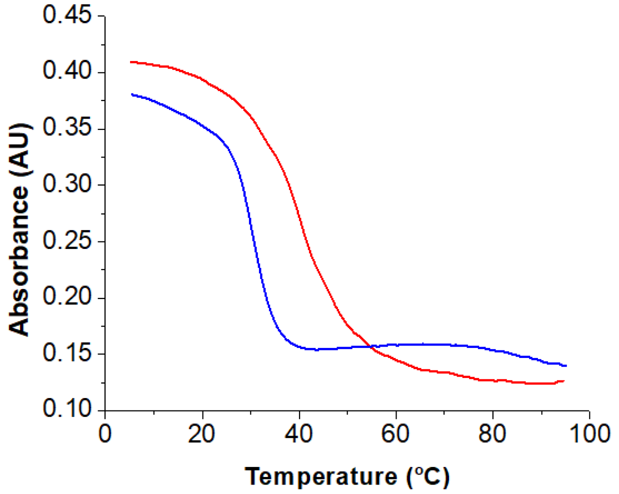

Figure S2G. Example UV melting (red) and annealing (blue) curves for d(TCCC)$_{12}$ at 2.5 μM in 10 mM sodium cacodylate with 100 mM KCl at pH 6.5.

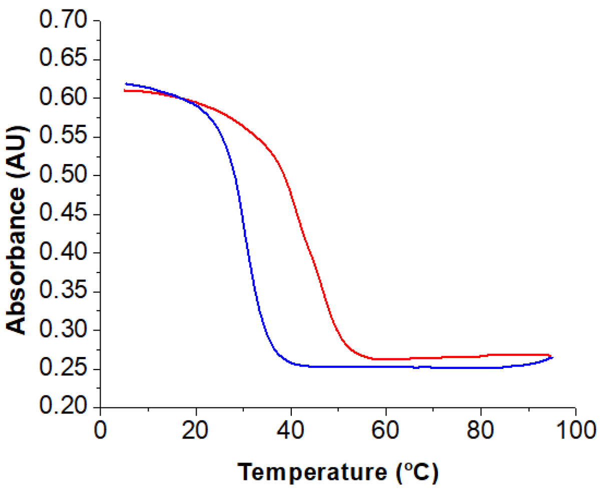

Figure S2H. Example UV melting (red) and annealing (blue) curves for d(TCCC)$_{14}$ at 2.5 μM in 10 mM sodium cacodylate with 100 mM KCl at pH 6.5.

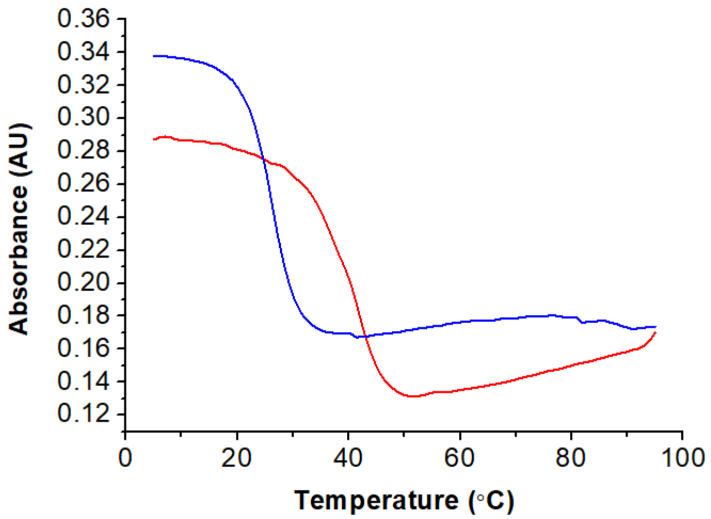

Figure S2I. Example UV melting (red) and annealing (blue) curves for d(TCCC)$_{15}$ at 2.5 μM in 10 mM sodium cacodylate with 100 mM KCl at pH 6.5.

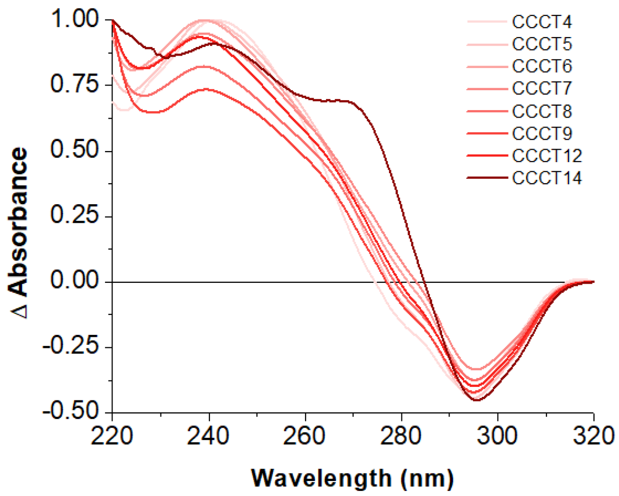

Figure S3. Thermal difference spectra for d(TCCC)$_{4-14}$ at 2.5 μM in 10 mM sodium cacodylate with 100 mM KCl at pH 6.5.

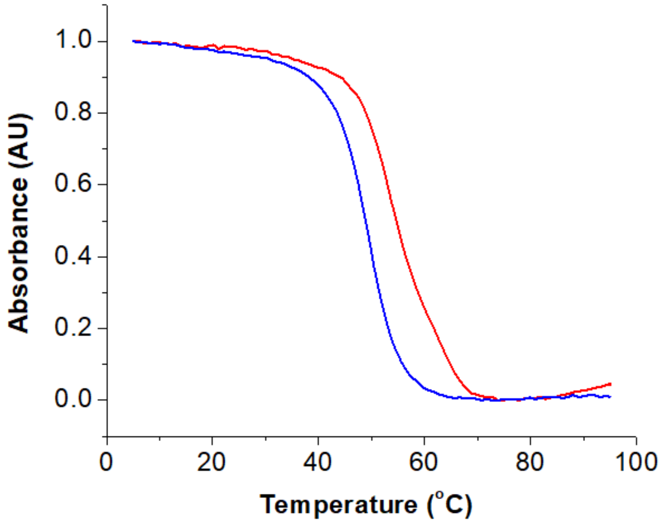

Figure S4A. Example UV melting (red) and annealing (blue) curves for (TCCC)$_5$ at 2.5 μM in 10 mM sodium cacodylate with 100 mM KCl at pH 5.3.

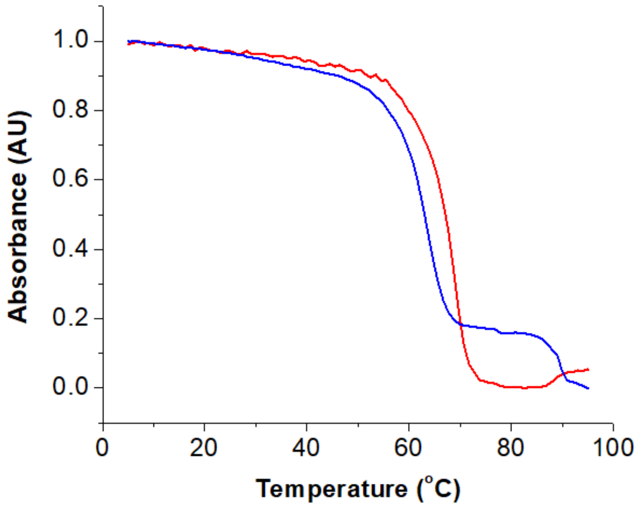

Figure S4B. Example UV melting (red) and annealing (blue) curves for $(TCCC)_9$ at 2.5 μM in 10 mM sodium cacodylate with 100 mM KCl at pH 5.3.

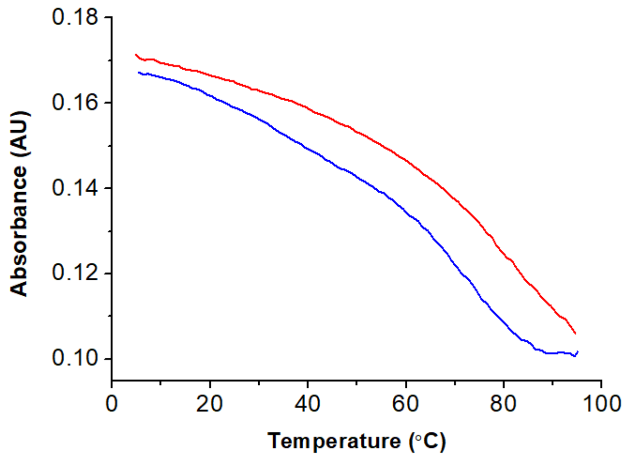

Figure S5A. Example UV melting (red) and annealing (blue) curves for $d(GGGA)_4$ at 2.5 μM in 10 mM sodium cacodylate with 20 mM KCl at pH 7.0.

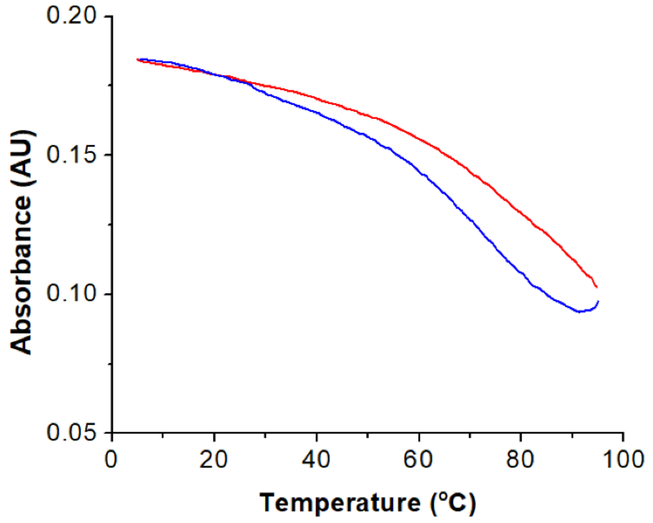

Figure S5B. Example UV melting (red) and annealing (blue) curves for $d(GGGA)_5$ at 2.5 μM in 10 mM sodium cacodylate with 20 mM KCl at pH 7.0.

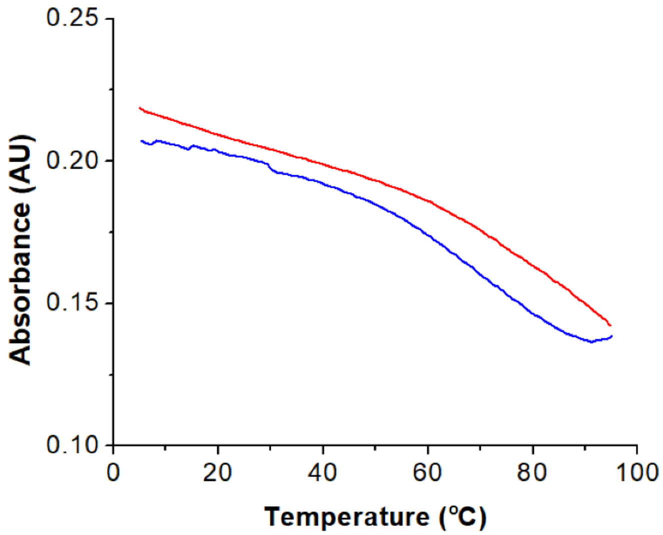

Figure S5C. Example UV melting (red) and annealing (blue) curves for d(GGGA)$_6$ at 2.5 μM in 10 mM sodium cacodylate with 20 mM KCl at pH 7.0.

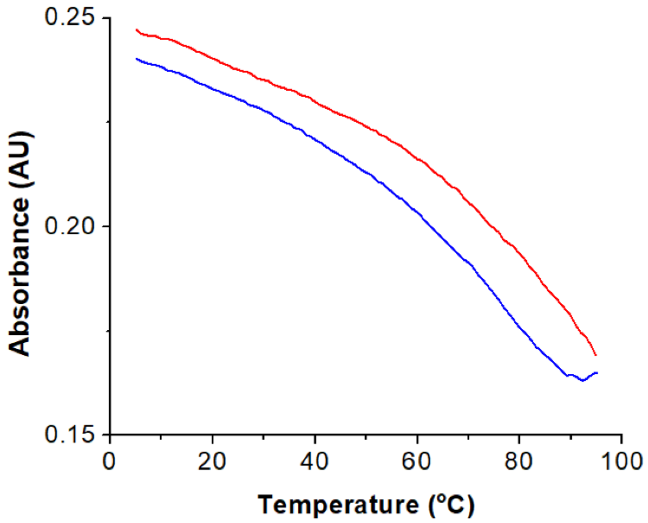

Figure S5D. Example UV melting (red) and annealing (blue) curves for d(GGGA)$_7$ at 2.5 μM in 10 mM sodium cacodylate with 20 mM KCl at pH 7.0.

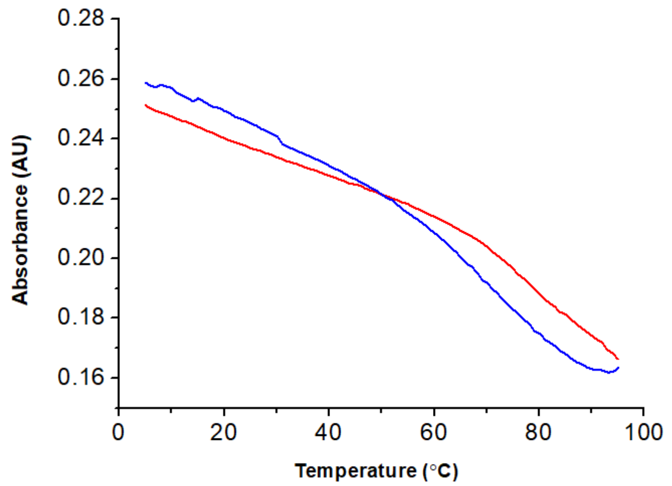

Figure S5E. Example UV melting (red) and annealing (blue) curves for d(GGGA)$_8$ at 2.5 μM in 10 mM sodium cacodylate with 20 mM KCl at pH 7.0.

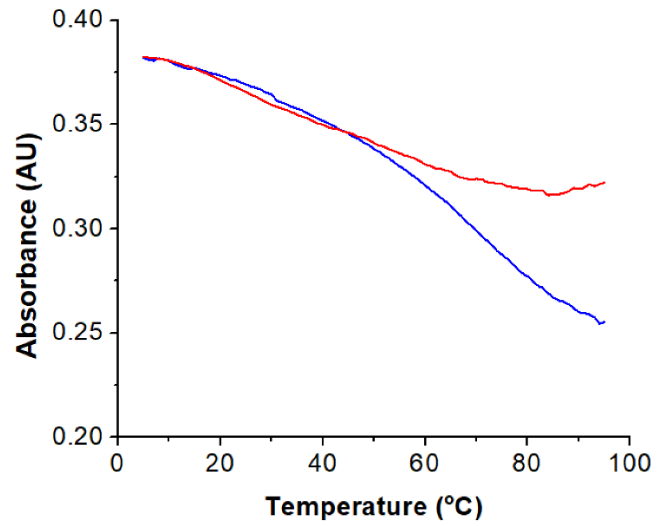

Figure S5F. Example UV melting (red) and annealing (blue) curves for d(GGGA)$_9$ at 2.5 μM in 10 mM sodium cacodylate with 20 mM KCl at pH 7.0.

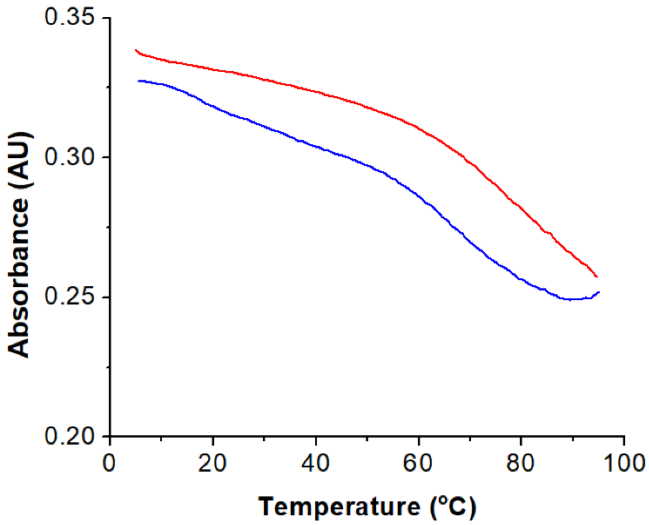

Figure S5G. Example UV melting (red) and annealing (blue) curves for $d(GGGA)_{12}$ at 2.5 μM in 10 mM sodium cacodylate with 20 mM KCl at pH 7.0.

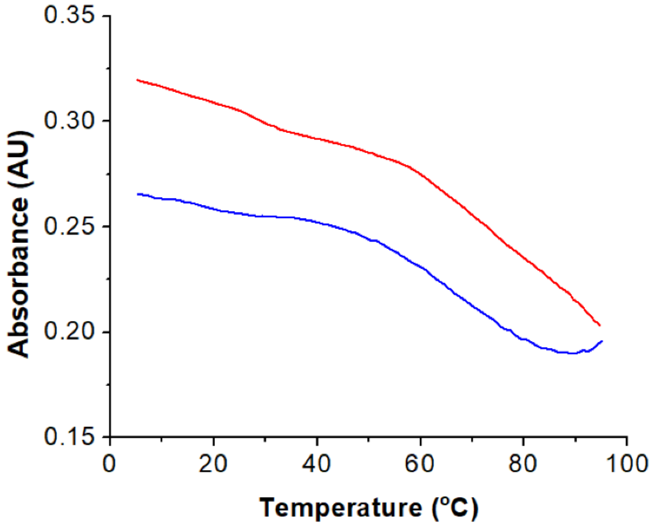

Figure S5H. Example UV melting (red) and annealing (blue) curves for $d(GGGA)_{14}$ at 2.5 μM in 10 mM sodium cacodylate with 20 mM KCl at pH 7.0.

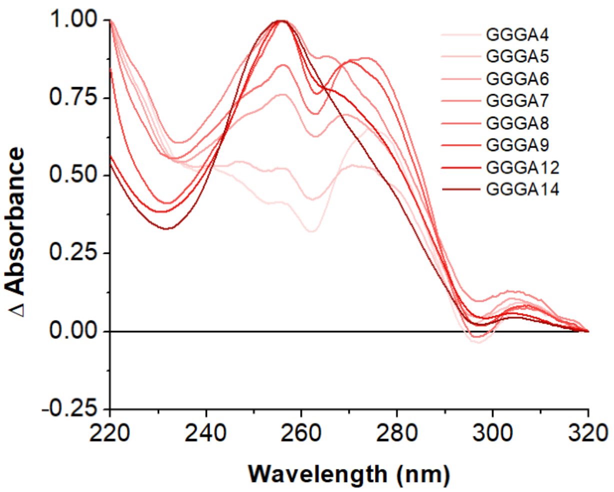

Figure S6. Thermal difference spectra for $(GGGA)_{4\text{-}14}$ at 2.5 μM in 10 mM sodium cacodylate with 100 mM KCl at pH 7.0.

Table S1. UV melting and annealing temperatures of GGGA repeats determined by the first derivative method. DNA at 2.5 μM in 10 mM sodium cacodylate with 20 mM KCl at pH 7.0. Values are the mean from duplicate or triplicate experiments with standard deviations. *Sample did not melt.

**Sequence**	**T_m /°C**	**T_a /°C**	**Hysteresis /°C**
**$(GGGA)_4$**	78.8 ± 2.5	71.9 ± 0.2	7.1 ± 2.5
**$(GGGA)_5$**	79.3 ± 4.3	72.5 ± 5.5	6.8 ± 2.0
**$(GGGA)_6$**	>95*	73.1 ± 2.6	21.9 ± 2.6
**$(GGGA)_7$**	>95*	69.4 ± 0.8	25.6 ± 0.8
**$(GGGA)_8$**	82.6 ± 9.5	73.4 ± 2.5	10.1 ± 6.7
**$(GGGA)_9$**	>95*	75.4 ± 2.9	19.6 ± 2.9
**$(GGGA)_{12}$**	78.3 ± 0.9	69.9 ± 4.8	7.2 ± 5.3
**$(GGGA)_{14}$**	>95*	71.9 ± 6.1	23.1 ± 6.1

Circular Dichroism Analysis of Length Variants

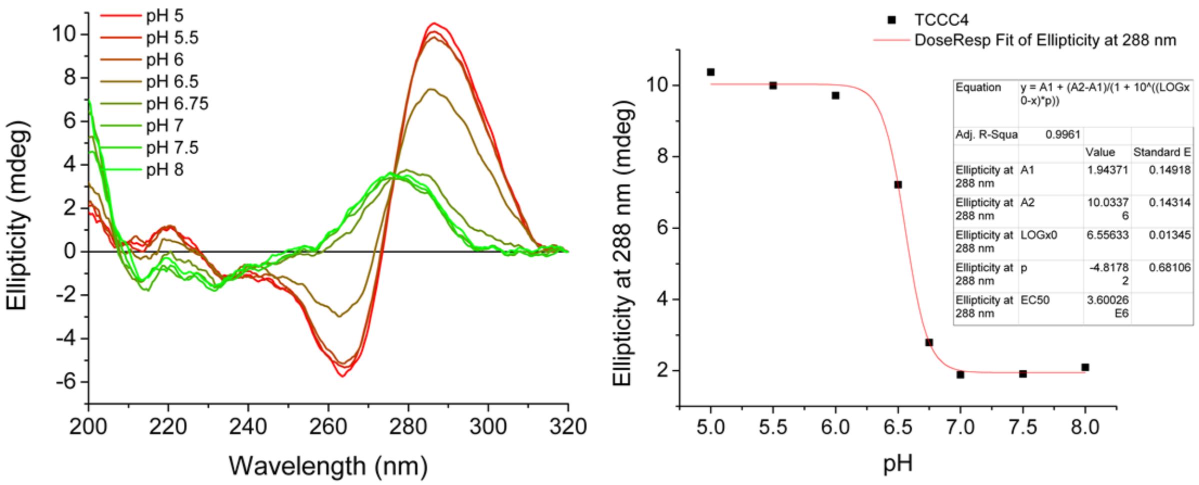

Figure S7A. CD spectra for $TCCC_4$. All oligonucleotides were diluted to a final concentration of 10 μM in 10 mM sodium cacodylate with 100 mM KCl at the respective pHs.

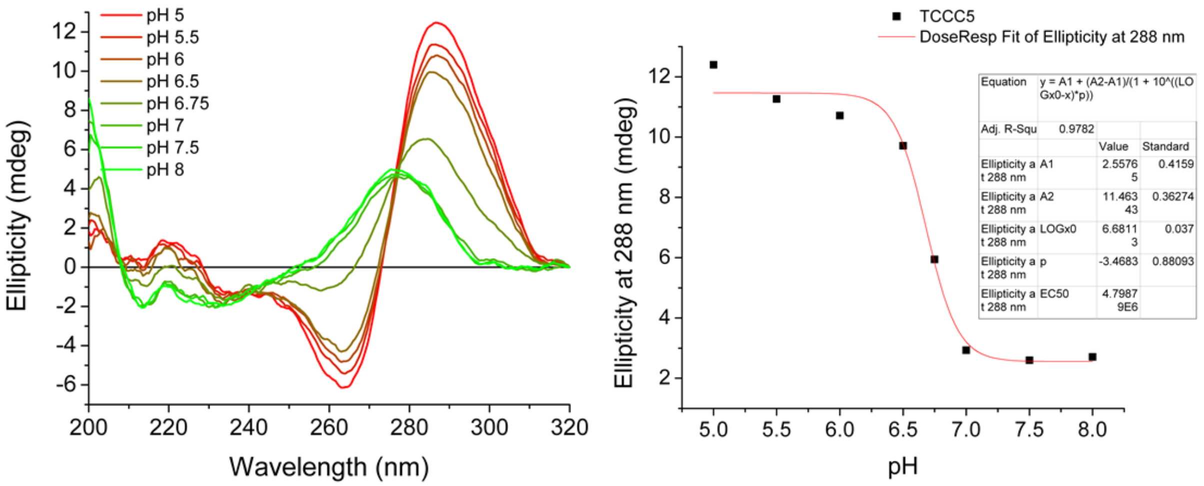

Figure S7B. CD spectra for $TCCC_5$. All oligonucleotides were diluted to a final concentration of 10 μM in 10 mM sodium cacodylate with 100 mM KCl at the respective pHs.

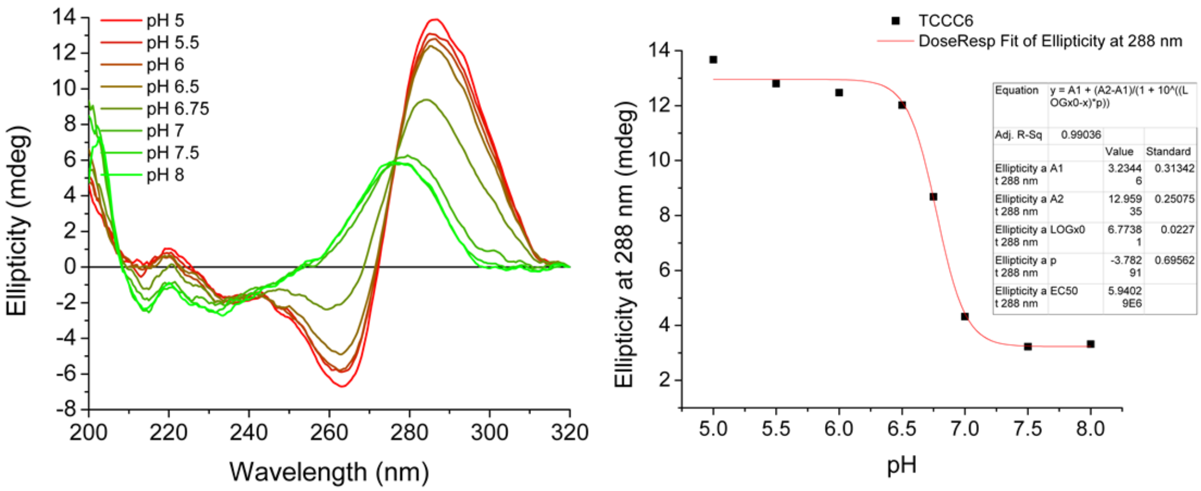

Figure S7C. CD spectra for TCCC$_6$. All oligonucleotides were diluted to a final concentration of 10 µM in 10 mM sodium cacodylate with 100 mM KCl at the respective pHs.

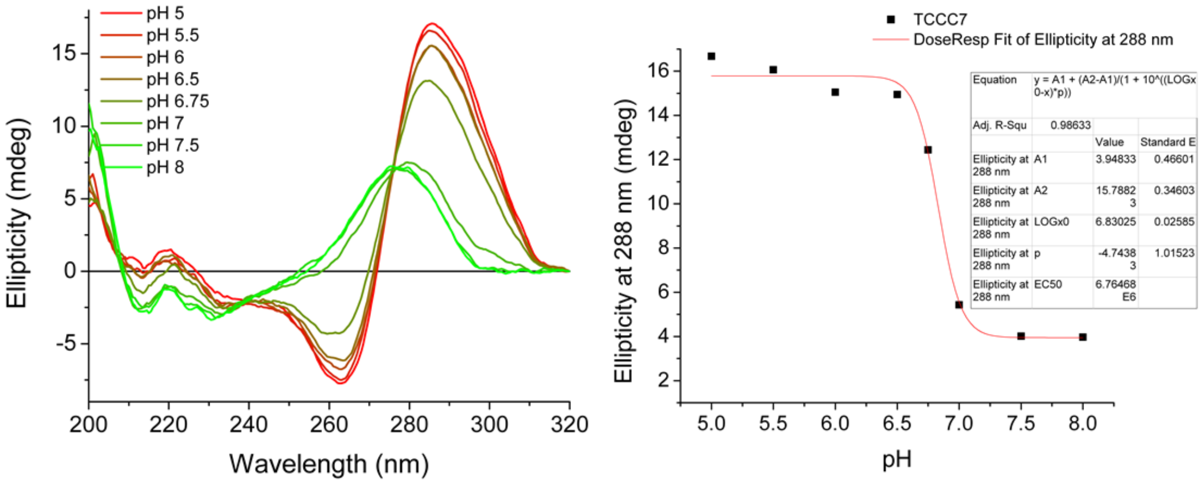

Figure S7D. CD spectra for TCCC$_7$. All oligonucleotides were diluted to a final concentration of 10 µM in 10 mM sodium cacodylate with 100 mM KCl at the respective pHs.

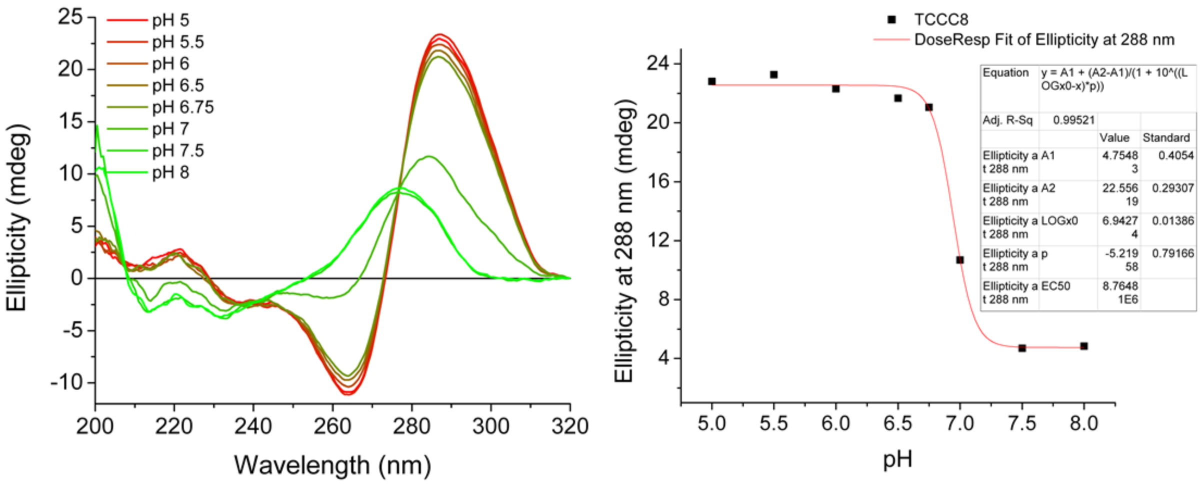

Figure S7E. CD spectra for $TCCC_8$. All oligonucleotides were diluted to a final concentration of 10 μM in 10 mM sodium cacodylate with 100 mM KCl at the respective pHs.

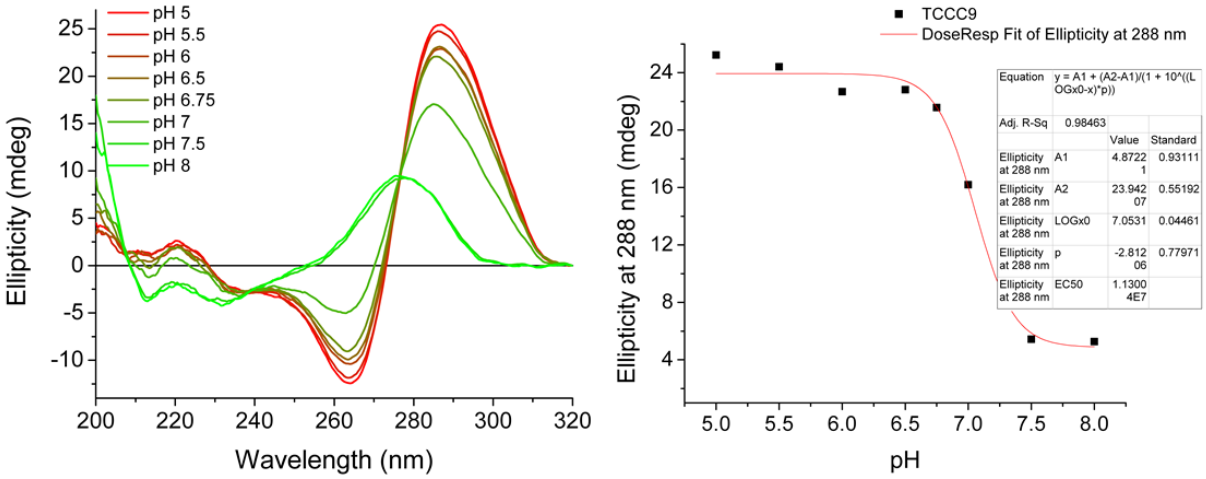

Figure S7F. CD spectra for $TCCC_9$. All oligonucleotides were diluted to a final concentration of 10 μM in 10 mM sodium cacodylate with 100 mM KCl at the respective pHs.

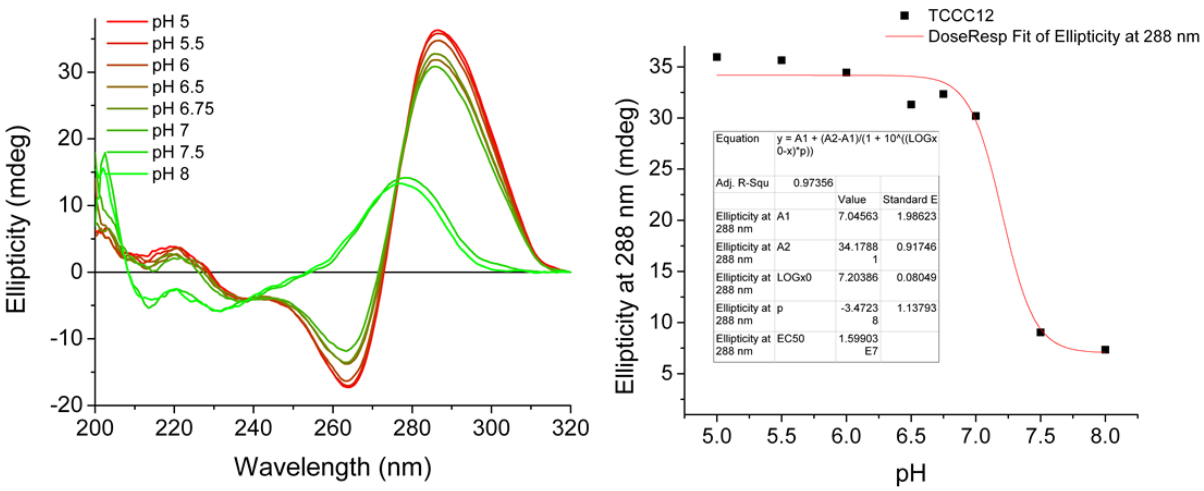

Figure S7G. CD spectra for $TCCC_{12}$. All oligonucleotides were diluted to a final concentration of 10 µM in 10 mM sodium cacodylate with 100 mM KCl at the respective pHs.

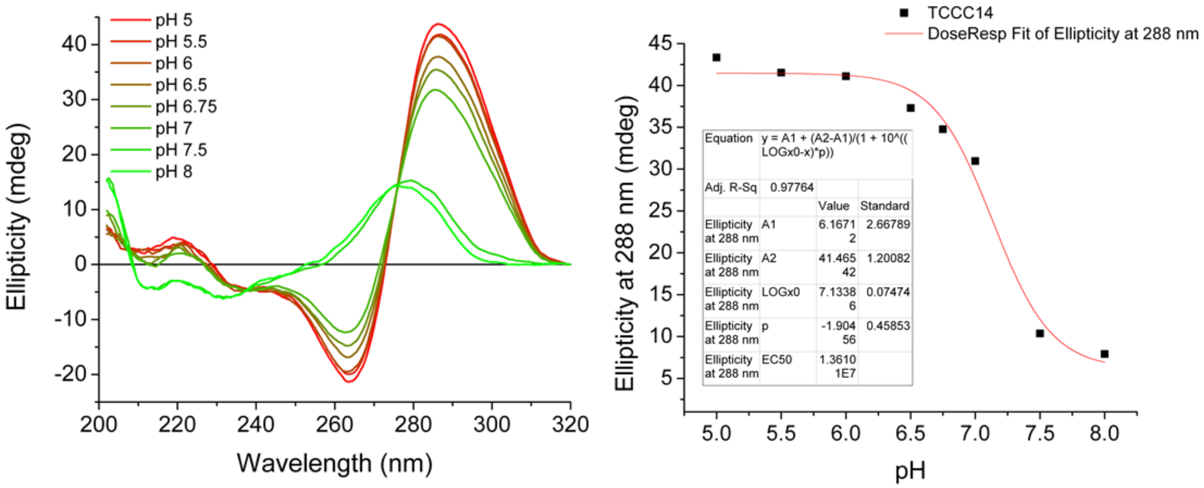

Figure S7H. CD spectra for $TCCC_{14}$. All oligonucleotides were diluted to a final concentration of 10 µM in 10 mM sodium cacodylate with 100 mM KCl at the respective pHs.

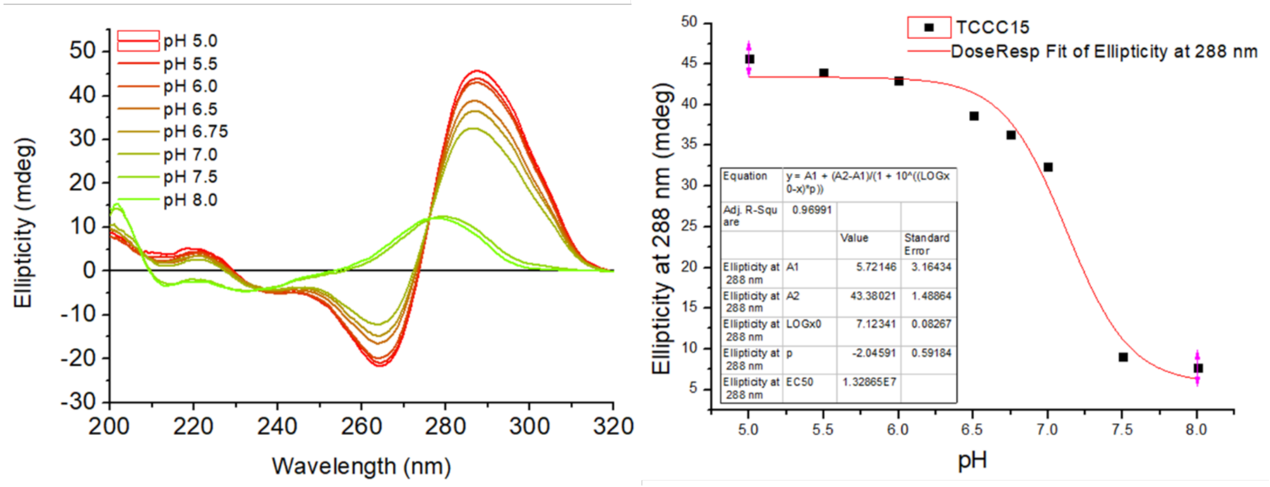

Figure S7I. CD spectra for $TCCC_{15}$. All oligonucleotides were diluted to a final concentration of 10 μM in 10 mM sodium cacodylate with 100 mM KCl at the respective pHs.

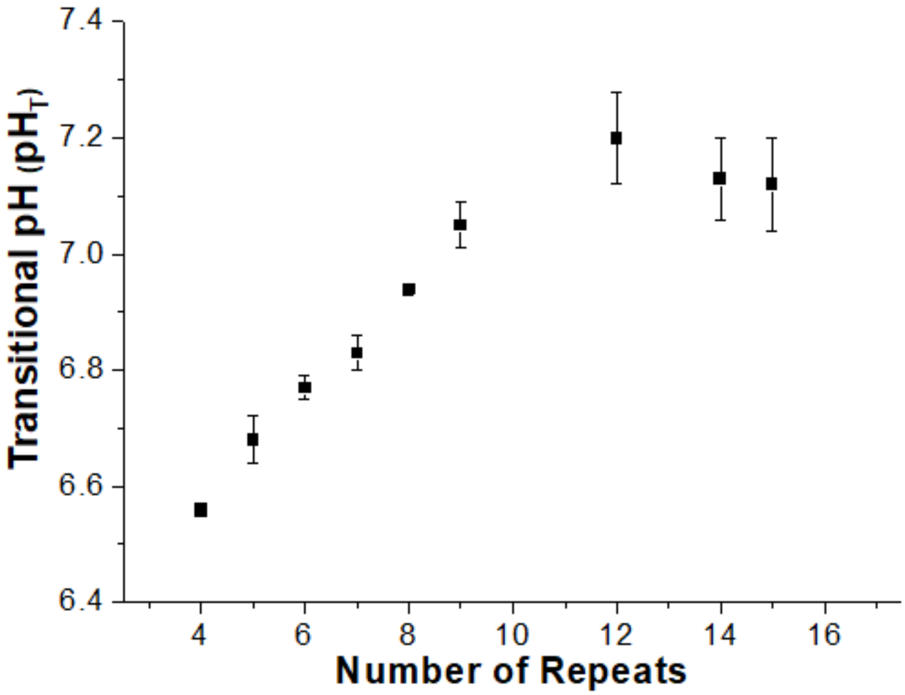

Figure S8. Plot of transitional pH against the number of CCCT repeats in the sequences. Error bars represent the error of the fit.

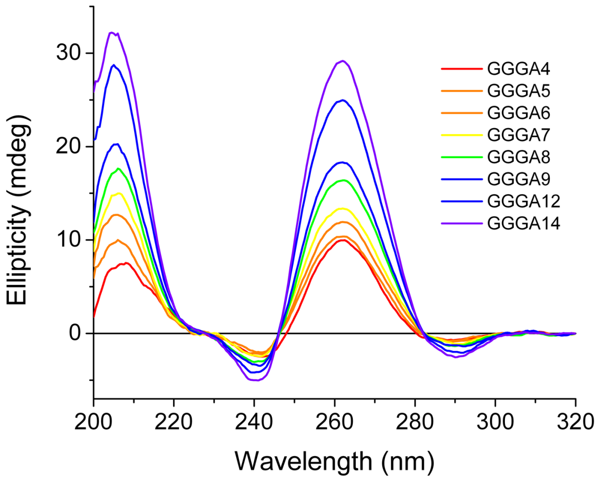

Figure S9. CD spectra for GGGA repeats. All oligonucleotides were diluted to a final concentration of 10 µM in 10 mM sodium cacodylate with 100 mM KCl at pH 7.0.
